# CRISPR screening using an expanded toolkit of autophagy reporters identifies TMEM41B as a novel autophagy factor

**DOI:** 10.1371/journal.pbio.2007044

**Published:** 2019-04-01

**Authors:** Christopher J. Shoemaker, Tina Q. Huang, Nicholas R. Weir, Nicole J. Polyakov, Sebastian W. Schultz, Vladimir Denic

**Affiliations:** 1 Department of Molecular and Cellular Biology, Harvard University, Northwest Labs, Cambridge, Massachusetts, United States of America; 2 Centre for Cancer Cell Reprogramming, Faculty of Medicine, University of Oslo, Oslo, Norway; 3 Department of Molecular Cell Biology, Institute for Cancer Research, Oslo University Hospital, Oslo, Norway; University of Oslo, Norway

## Abstract

The power of forward genetics in yeast is the foundation on which the field of autophagy research firmly stands. Complementary work on autophagy in higher eukaryotes has revealed both the deep conservation of this process, as well as novel mechanisms by which autophagy is regulated in the context of development, immunity, and neuronal homeostasis. The recent emergence of new clustered regularly interspaced palindromic repeats/CRISPR-associated protein 9 (CRISPR/Cas9)-based technologies has begun facilitating efforts to define novel autophagy factors and pathways by forward genetic screening in mammalian cells. Here, we set out to develop an expanded toolkit of autophagy reporters amenable to CRISPR/Cas9 screening. Genome-wide screening of our reporters in mammalian cells recovered virtually all known autophagy-related (ATG) factors as well as previously uncharacterized factors, including vacuolar protein sorting 37 homolog A (VPS37A), transmembrane protein 251 (TMEM251), amyotrophic lateral sclerosis 2 (ALS2), and TMEM41B. To validate this data set, we used quantitative microscopy and biochemical analyses to show that 1 novel hit, TMEM41B, is required for phagophore maturation. TMEM41B is an integral endoplasmic reticulum (ER) membrane protein distantly related to the established autophagy factor vacuole membrane protein 1 (VMP1), and our data show that these two factors play related, albeit not fully overlapping, roles in autophagosome biogenesis. In sum, our work uncovers new ATG factors, reveals a malleable network of autophagy receptor genetic interactions, and provides a valuable resource (http://crispr.deniclab.com) for further mining of novel autophagy mechanisms.

## Author summary

Eukaryotic cells use autophagy to eliminate unwanted structures—such as protein aggregates, intracellular pathogens, and damaged organelles—that are too large to be handled by the proteasome. This unusual vesicle transport pathway begins with packaging of cytoplasmic targets into a double-membrane vesicle (autophagosome) and ends with their degradation in the lysosome. A deeper understanding of autophagy as a regulated mechanism for protein turnover has the potential to provide new therapies for diverse human pathologies, including neurodegenerative disease, cardiometabolic disease, and cancer. Here, we set out to build new reporters for studying mammalian autophagy by genetic screening. This approach allowed us to carry out pooled genome-wide clustered regularly interspaced palindromic repeats (CRISPR) knockout screens and recover virtually all known mammalian autophagy-related (ATG) factors. In addition, we uncovered several uncharacterized proteins, including the endoplasmic reticulum (ER) transmembrane protein 41B (TMEM41B), which we went on to show is required for normal growth of autophagosome precursor membranes around their targets. More broadly, our data set provides a comprehensive resource of genes that affect autophagosome trafficking to the lysosome for further hypothesis testing.

## Introduction

Autophagy is an umbrella term for a broad family of trafficking pathways that transport cytoplasmic material to the lysosome for destruction. Its most studied form, macroautophagy (hereafter “autophagy”), involves the formation of a double-membrane vesicle (the autophagosome) that sequesters cytoplasm and delivers it to the lysosome by vesicle fusion. The first factors identified in autophagosome biogenesis, the so-called autophagy-related (ATG) factors, were originally identified by genetic screens in yeast [1–3]. This list eventually matured to its current state that includes approximately 40 yeast ATGs and distinguishes “core” factors required for all forms of autophagy from those required only for specific substrates (e.g., a damaged mitochondrion) [4]. The inventory of factors involved in autophagy has been further expanded by the discovery of additional autophagy factors not found in yeast, such as ATG101, EPG5, and vacuole membrane protein 1 (VMP1) [5–8]. Whether additional autophagy factors have yet to be discovered remains an open question.

The molecular functions of ATGs in autophagosome biogenesis are still being delineated. Initiation involves construction of a cup-shaped precursor membrane (the phagophore) in close apposition to the endoplasmic reticulum (ER) [9–13]. This process is dependent on the RB1 inducible Coiled-Coil 1 (RB1CC1) scaffolding protein and post-Golgi vesicles containing ATG9A [14–16]. Next, recruitment of an autophagy-specific phosphoinositide-3 kinase (PI3K) complex results in localized phosphatidylinositol-3 phosphate (PI3P) formation and recruitment of PI3P effectors such as WD repeat domain, phosphoinositide interacting 2 (WIPI2) [17,18]. Nascent phagophores are then elongated concurrent with lipid conjugation of microtubule-associated protein 1 light chain 3B (LC3) family proteins (autophagy-related protein 8 [Atg8] in yeast), although the requirement for LC3 lipidation has recently been challenged [19–21].

Efficient encapsulation of specific cell material within autophagosomes is mediated by autophagy receptors, adaptor proteins whose defining feature is their ability to bridge cargo with lipidated LC3 present on the autophagosomal membrane [22]. Interactions between cargos, receptors, and lipidated LC3 leads to their mutual capture by autophagosomes and subsequent lysosomal destruction. This feature of LC3 has been exploited to establish the most widely used reporter of autophagic flux: tandem-fluorescent (tf) LC3 (tfLC3). In this approach, 2 fluorescent protein tags (red fluorescent protein [RFP] and green fluorescent protein [GFP]) are appended to the amino terminus of LC3. Upon lysosomal delivery of tfLC3, low pH conditions within the lysosomal lumen selectively quench GFP fluorescence, resulting in a dramatic increase in the observed Red:Green fluorescence ratio [23,24]. In a similar manner, tf sequestosome 1 (SQSTM1) has been used to measure the flux of this heavily studied autophagy receptor [24].

Here, we further expanded this approach to include 3 additional SQSTM1-like receptors (nuclear dot protein 52 [NDP52], tax1 binding protein 1 [TAX1BP1], and neighbor of BRCA1 gene 1 [NBR1]) broadly implicated in selective autophagy. With this expanded toolkit in hand, we performed genome-wide clustered regularly interspaced palindromic repeats (CRISPR) screens to identify selective autophagy factors in mammalian cells. We recovered virtually all known ATG factors and uncovered several uncharacterized proteins for further study. We validated our list using quantitative cell microscopy and biochemical analyses to define transmembrane protein 41B (TMEM41B) as an integral ER membrane protein that is required for phagophore maturation in higher eukaryotes. TMEM41B shares a broadly conserved transmembrane domain (pfam09335) with an established autophagy factor, VMP1, and our data show that these 2 factors play related, though not fully overlapping, roles in autophagosome biogenesis.

## Results

### Expanding the toolkit of autophagic flux reporters for genetic screening

**Fig 1:**
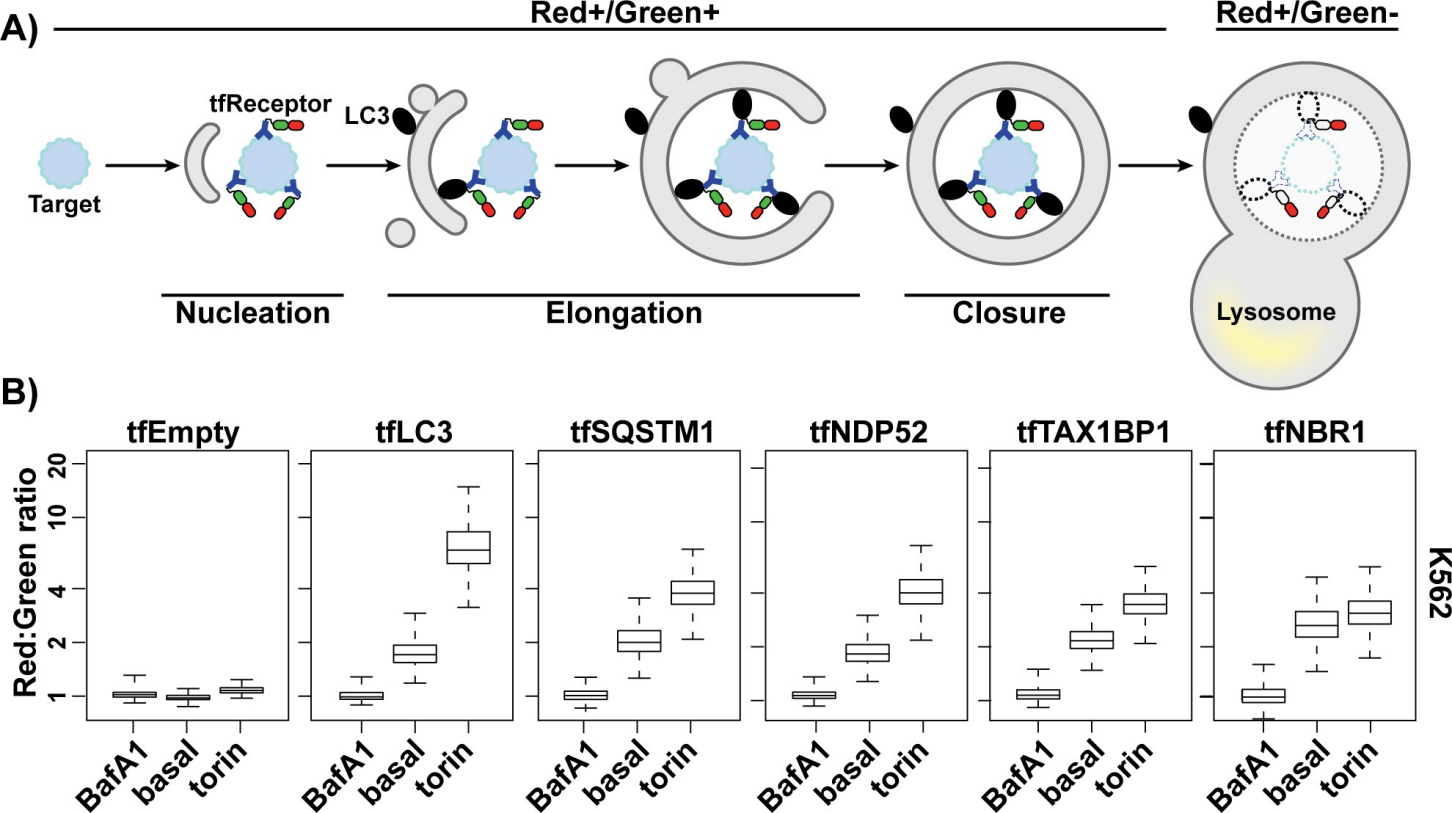
An expanded toolkit of autophagy reporters performs comparably to tfLC3. (A) Schematic depicting how tfReceptors measure autophagic flux. tfReceptors bind to targets and interface with additional components of the autophagy machinery such as lipidated LC3 (black ovals). GFP fluorescence is selectively quenched in low pH environments, leading to an increased Red:Green ratio upon exposure of tfReceptor to the lumen of the lysosome. Dashed lines indicate degradation by lysosomal hydrolases. (B) K562 cells expressing the indicated tf proteins from the AAVS1 locus were analyzed by flow cytometry under basal conditions and after 18 h treatment with 100 nM BafA1 or 250 nM torin. Values for each reporter were normalized to BafA1 treatment. Plots show median Red:Green ratios, inner quartiles (boxed regions), and 10th and 90th percentile (whiskers). *n* > 1,000 cells for each sample. Related to S1 Fig. Underlying data for all summary statistics can be found in S1 Data. AAVS1, adeno-associated virus integration site 1; BafA1, Bafilomycin A1; GFP, green fluorescent protein; LC3, microtubule-associated protein 1 light chain 3B; NBR1, neighbor of BRCA1 gene 1; NDP52, nuclear dot protein 52; SQSTM1, sequestosome 1; TAX1BP1, tax 1 binding protein 1; tf, tandem-fluorescent.

K562 cells (a human myelogenous leukemia line) can be readily cultured in cell suspension, which has led to their frequent use in pooled CRISPR screens. To devise a general strategy for monitoring autophagic flux in K562 cells, we began by constructing 6 homologous gene cassettes: 1 (tfLC3) encoding an N-terminal, tf (RFP-GFP) fusion with LC3B, 4 (tfSQSTM1, tfNDP52, tfTAX1BP1, and tfNBR1) with N-terminal fusions to SQSTM1-related autophagy receptors, and 1 (tfEmpty) as a negative control (S1A Fig). The RFP to GFP fluorescence ratio (Red:Green ratio) of tfLC3 is a widely used metric for autophagic flux predicated on the selective quenching of GFP in low pH environments, such as the lysosomal lumen (Fig 1A). Following cassette integration at the adeno-associated virus integration site 1 (AAVS1) locus, we analyzed cells by flow cytometry under basal conditions and following treatment with either Bafilomycin A1 (BafA1), an inhibitor of autophagosome–lysosome fusion, or torin, a small-molecule inducer of autophagy. Corresponding changes in the observed Red:Green ratio of tfLC3 and tfReceptors provided strong evidence for both basal and induced autophagy in K562 cells (Fig 1B). By contrast, the Red:Green ratio of tfEmpty cells was unresponsive to either drug treatment, arguing that nonselective reporter engulfment makes a negligible contribution to our flux measurements. We obtained similar results using adherent human embryonic kidney HEK293T cells (S1B Fig). Furthermore, we confirmed that torin-induced increases in Red:Green ratio were due to selective GFP quenching (S1C Fig) and that both basal and induced autophagy were dependent on the known autophagy-related factors RB1CC1 and ATG13 (S1D and S1E Fig). Collectively, these data validated a broad panel of autophagic flux reporters for subsequent use as genetic screening tools in K562 cells.

### CRISPR screens for modifiers of autophagic flux recover known ATG factors and reveal new candidates

**Fig 2:**
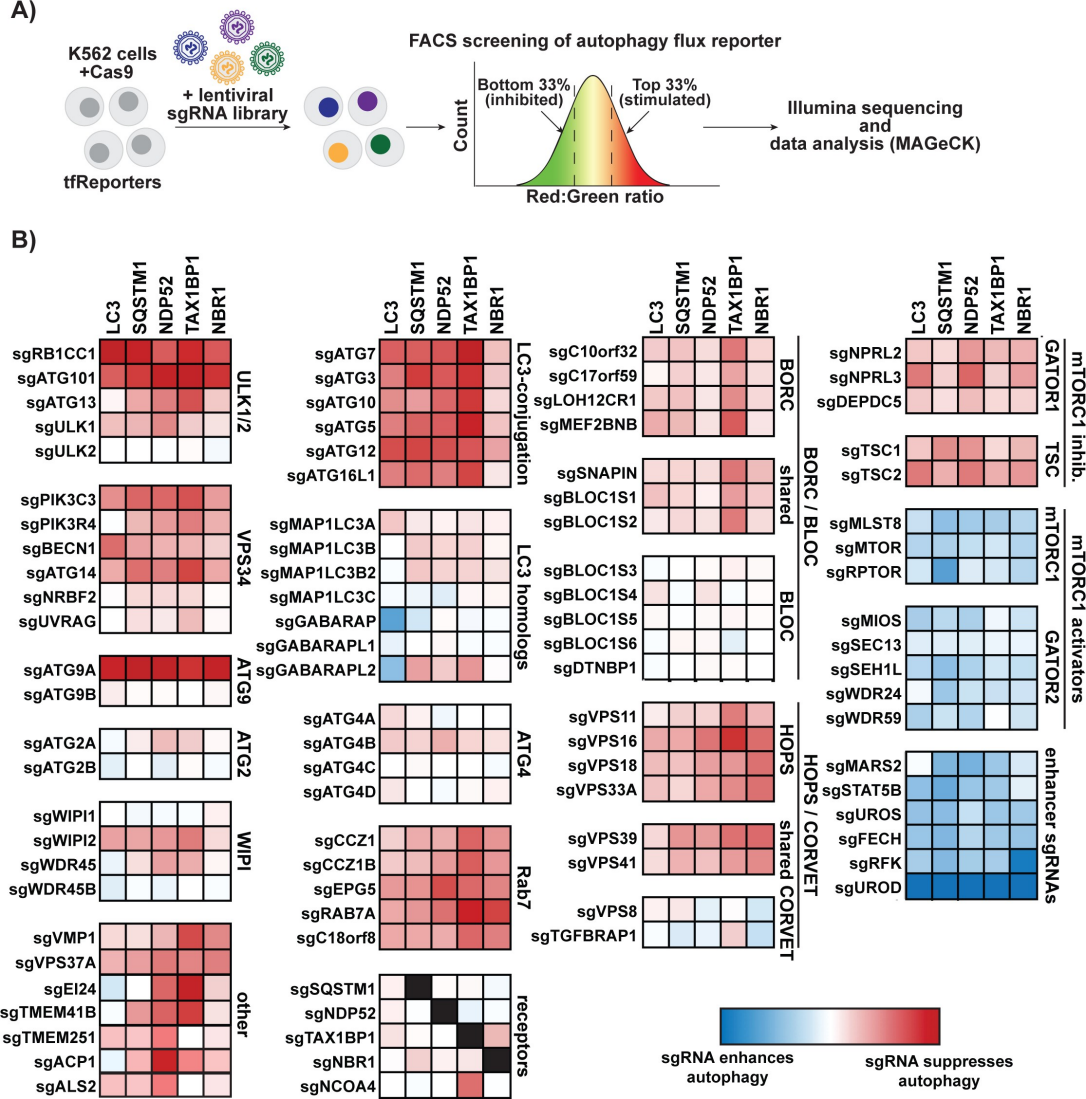
Genome-wide CRISPR screening using tfReporters reveals known and novel regulators of basal autophagy. (A) Schematic depicting a pooled CRISPR screening strategy for defining genetic modifiers of tfReporters (tfLC3 and tfReceptors) under basal conditions. K562 cells expressing Cas9 were transduced with the Brunello lentiviral sgRNA library. The top and bottom third of cells were collected based on Red:Green ratio. sgRNA sequences from collected cells were obtained by Illumina sequencing and analyzed by MAGeCK. (B) Shown is a heat map of averaged beta scores (similar to fold-enrichment) for the indicated sgRNAs. Scores are color coded based on the effect of the indicated sgRNA (red, sgRNA suppresses autophagy; blue, sgRNA enhances autophagy). For comparison, known ATG factors (regardless of effect size) are presented alongside novel hits. Genes are clustered loosely based on their known function. sgRNAs listed under “other” (e.g., TMEM41B) were chosen from targets whose beta scores placed them in the top approximately 50 hits for 1 or more reporters. sgRNAs listed under “enhancer sgRNAs” target the 6 genes that scored better than the highest-scoring known autophagy regulator, MLST8. Underlying read counts and beta scores are presented in S2 Data and S3 Data, respectively. An interactive interface for data is also available online at http://crispr.deniclab.com. ATG, autophagy-related; Cas9, CRISPR-associated protein 9; CRISPR, clustered regularly interspaced palindromic repeats; FACS, fluorescence activated cell sorting; LC3, microtubule-associated protein 1 light chain 3B; MAGeCK, model-based analysis of genome-wide CRISPR-Cas9 knockout; MLST8, MTOR associated protein LST8 homolog; sgRNA, single guide RNA; tf, tandem-fluorescent; TMEM41B, transmembrane protein 41B; VMP1, vacuole membrane protein 1.

Next, we performed a series of genome-wide, pooled CRISPR knockout screens in K562 cells co-expressing CRISPR-associated protein 9 (Cas9) with each of our autophagic flux reporters. To this end, we utilized the Brunello single guide RNA (sgRNA) library containing 76,441 sgRNAs covering 19,114 genes [25]. Following transduction of the sgRNA library, we used fluorescence activated cell sorting (FACS) to collect the top and bottom third of cells ranked on the basis of their Red:Green ratio (Fig 2A). Read counts of sgRNAs derived from these cell fractions were obtained by Illumina sequencing and computationally analyzed by model-based analysis of genome-wide CRISPR-Cas9 knockout (MAGeCK) [26,27]. The resulting output for each gene includes a beta score (similar to log-fold change) as a proxy for its strength as an autophagy effector (Fig 2B). To facilitate public mining of these data, we have made them freely available using an interactive interface at http://crispr.deniclab.com.

From the global analysis of gene hits (Fig 2B), we made multiple observations that validated our genetic screening approach. First, virtually all known ATG factors were identified as required for reporter flux. Second, we observed the expected phenotype for positive and negative regulators of mammalian target of rapamycin complex 1 (mTORC1), a potent inhibitor of autophagy signaling. Third, we could distinguish protein complexes implicated in autophagy from related complexes with shared subunits. For example, our analysis distinguished the autophagic role of the homotypic fusion and vacuole protein sorting (HOPS) tethering complex from the related class C core vacuole/endosome tethering (CORVET) complex. Similarly, hits in the BLOC-one-related complex (BORC) disrupted autophagy, but we found no hits among subunits specific to the related biogenesis of lysosome-related organelles complex (BLOC-1) (Fig 2B).

**Fig 3:**
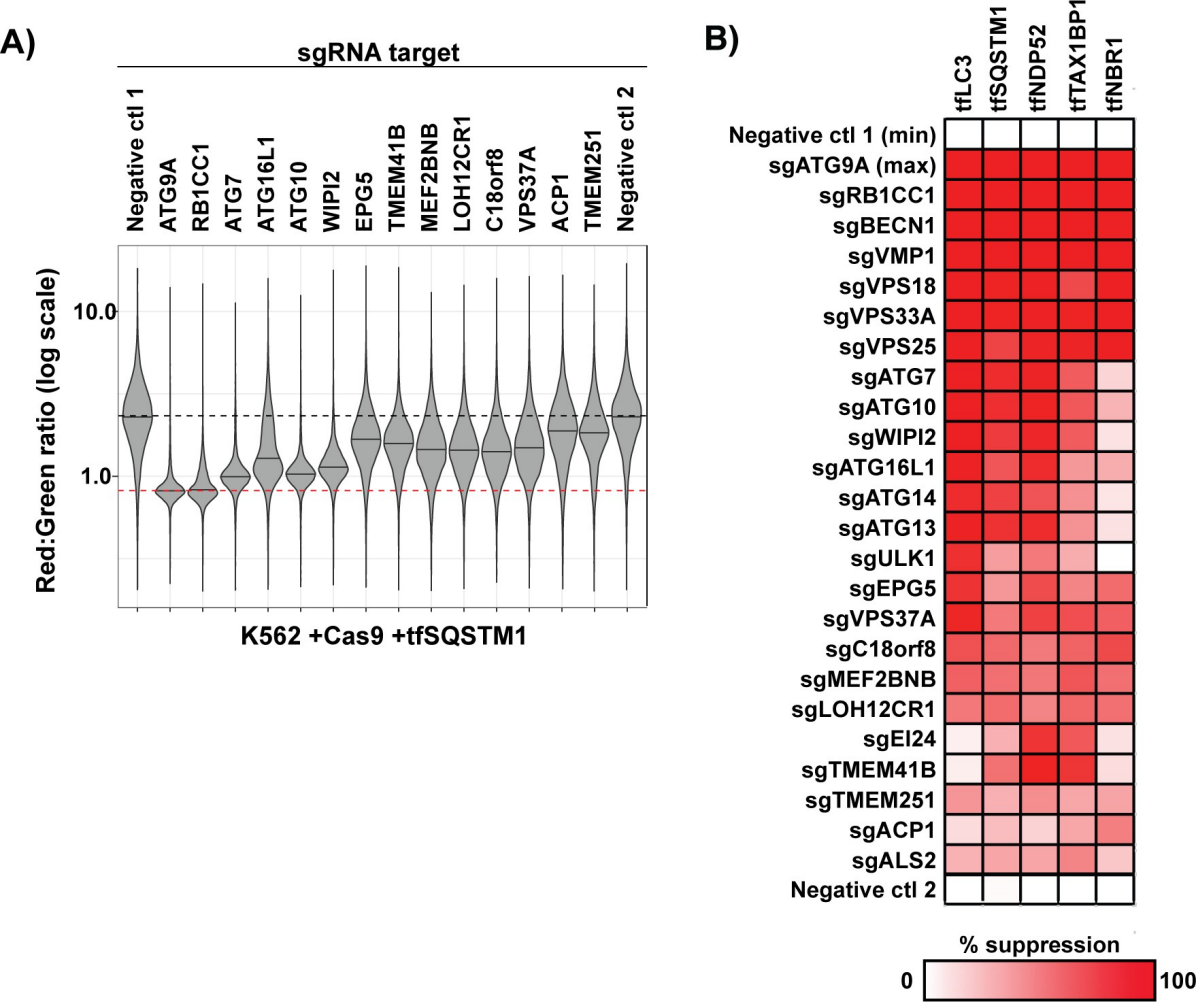
Novel screen hits robustly yield autophagy phenotypes in single gene knockout experiments. (A) K562 cells co-expressing Cas9 and tfSQSTM1 were transduced with individual sgRNAs against the indicated genes or with nontargeting sgRNA controls. Cells were pretreated with torin to maximize the dynamic range of tfSQSTM1 signal and analyzed by flow cytometry (*n* > 5,000 cells). Shown are violin plots generated in R using ggplot2. Median values for each sample are identified by a black line. Bimodal populations were deconvolved in an unbiased manner using the BifurGate function of FlowJo. The black dotted line across all samples corresponds to the Red:Green ratio of unaffected cells (i.e., negative ctl 1). The red dotted line across all samples corresponds to the ratio observed under maximally inhibited conditions (sgATG9A). See S2A–S2D Fig for similar data from the remaining tfReporters. (B) Heat map indicating the phenotypic strengths of indicated gene knockouts derived from the primary data in part A and S2A–S2D Fig. The median Red:Green ratio of each population was used to calculate the fold repression according to the following formula: (ratio_sgGene_ − ratio_sgATG9A_) ÷ (ratio_sgControl_ − ratio_sgATG9A_), with the assumption that sgATG9A yields a true autophagy-null phenotype. Deeper shades of red indicate stronger inhibitory phenotypes. Black boxes indicate unscored cases. Related to S2 Fig. Underlying data for all summary statistics can be found in S1 Data. ACP1, acid phosphatase 1; ATG, autophagy-related; Cas9, CRISPR-associated protein 9; ctl, control; EPG5, ectopic P-granules autophagy protein 5 homolog; LOH12CR1, loss of heterozygosity 12 chromosomal region 1; MEF2BNB, MEF2B neighbor; RB1CC1, RB1 inducible coiled-coil 1; sgRNA, single guide RNA; SQSTM1, sequestosome 1; tf, tandem-fluorescent; TMEM251, transmembrane protein 251; TMEM41B, transmembrane protein 41B; VPS37A, vacuolar protein sorting 37 homolog A; WIPI2, WD repeat domain, phosphoinositide interacting 2.

Our screening approach also identified several uncharacterized factors as strong modifiers of autophagy. To examine these hits further, we retested them as individual sgRNAs alongside a broad panel of known ATG factors (Fig 3A; S2A–S2D Fig). Indeed, the vast majority of individual sgRNAs increased or decreased flux proportionate to their beta score ranking (compare Fig 3B and Fig 2B). By contrast, we found that sgRNAs targeting uroporphyrinogen decarboxylase (UROD) were spurious hits that modified the Red:Green ratio by drastically enhancing RFP fluorescence (S2E Fig). We also confirmed that tfNBR1 flux in K562 cells was largely independent of ATG factors required for LC3 lipidation (e.g., ATG7), which agrees with our original screen findings (Fig 3B). ATG7-independent autophagosome formation has been reported in other cell types, and our work suggests that NBR1 might provide a useful new marker for further dissecting this process [28–30]. In sum, our genetic screening strategy and hit validation robustly uncovered known ATG factors and unveiled new candidates for further study.

### TMEM41B is a conserved ER membrane protein required for autophagy

TMEM41B stood out among our uncharacterized hits because of its strong phenotype (particularly for tfNDP52 and tfTAX1BP1) in K562 cells. This protein is predicted to span the membrane 6 times and to carry a di-lysine, C-terminal Golgi-to-ER retrieval signal (S3A Fig) [31]. In line with these predictions, an unbiased proteomic analysis of subcellular fractions previously assigned TMEM41B’s residence to the ER [32]. Indeed, when we immunoprecipitated TMEM41B we found that N-terminally tagged, but not C-terminally tagged, TMEM41B co-immunoprecipitated a coat protein I (COPI) complex component of Golgi-to-ER vesicles from detergent-solubilized cell lysates (S3B Fig). Furthermore, when we tagged the N-terminus of endogenous TMEM41B with the 11th beta strand of GFP (GFP11) and co-expressed a complementary GFP fragment (beta strands 1 through 10 [GFP1–10]), we observed a reticular GFP fluorescence signal that colocalized with the ER marker calnexin (S3C Fig).

**Fig 4:**
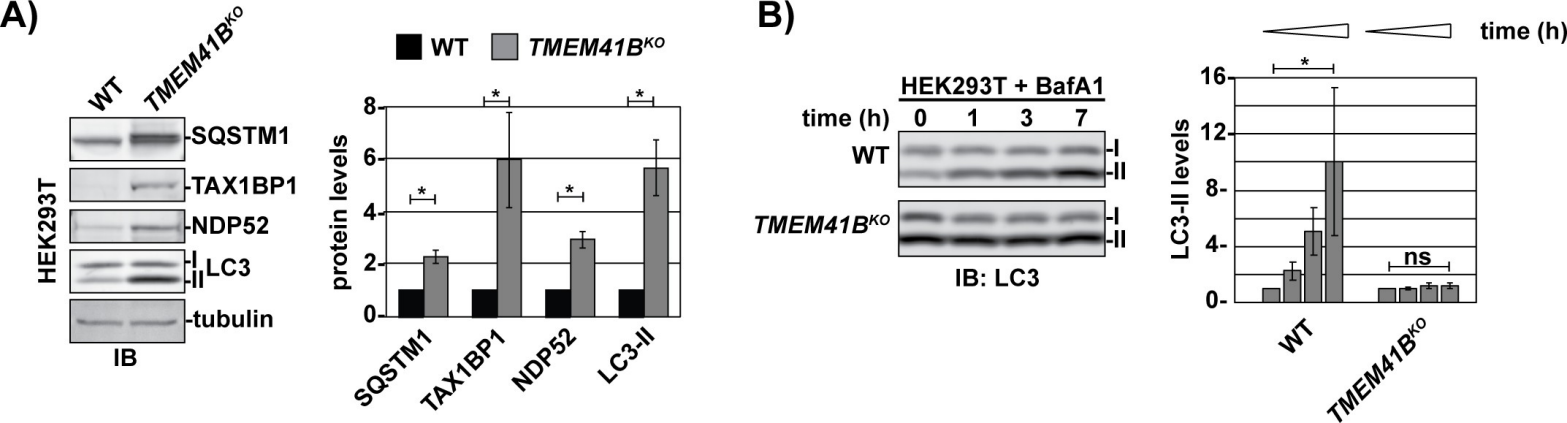
TMEM41B is required for autophagic flux. (A) Extracts derived from WT and *TMEM41B*^*KO*^ HEK293T cells were resolved by SDS-PAGE followed by IB with indicated antibodies. All samples were normalized by total protein using a BCA assay prior to loading. I and II indicate the unmodified and lipidated forms of LC3. Bar graphs show the mean ± SD of each sample from 3 independent experiments. Protein levels in WT cells were normalized to 1; *p*-values were determined using a one-sample *t* test. *p < 0.05. (B) WT and *TMEM41B*^*KO*^ HEK293T knockout cells were treated with 250 nM BafA1 for the times indicated. The corresponding cell extracts were normalized by total protein, resolved by SDS-PAGE, and analyzed by IB for LC3. I and II indicate the unmodified and lipidated forms of LC3. Bar graphs show the mean ± SD of each sample from 3 independent experiments; *p*-values were determined using a one-sample *t* test. *p < 0.05. Related to S3 Fig. Underlying data for all summary statistics can be found in S1 Data. BafA1, Bafilomycin A1; BCA, bicinchoninic acid; HEK, human embryonic kidney; IB, immunoblotting; LC3, microtubule-associated protein 1 light chain 3B; NDP52, nuclear dot protein 52; ns, not significant; SQSTM1, sequestosome 1; TAX1BP1, tax 1 binding protein 1; TMEM41B, transmembrane protein 41B; WT, wild type.

To test whether TMEM41B is critical for autophagy in other human cell lines, we knocked out its gene in HEK293T and HCT116 cells, where we observed even more robust effects on autophagy than in our original K562 background. Specifically, immunoblotting (IB) analysis of endogenous SQSTM1, TAX1BP1, NDP52, and LC3 revealed their accumulation in the absence of TMEM41B consistent with the possibility of reduced autophagic turnover (Fig 4A; S3D and S3E Fig). Two complementary measures of autophagic flux further support this interpretation. First, treatment of *TMEM41B*^*KO*^ cells with BafA1 was unable to induce further accumulation of lipidated LC3 (LC3-II) (Fig 4B). Second, tfLC3 and tfSQSTM1 flux were strongly repressed in *TMEM41B*^*KO*^ cells, comparable to *ATG7*^*KO*^ control cells (S3F and S3G Fig). Taken together, these data argue that autophagic flux is substantially inhibited in the absence of TMEM41B.

### TMEM41B mediates phagophore maturation

While autophagy was dramatically impeded in the absence of *TMEM41B*, it was unclear whether this defect was caused by the inability to properly initiate autophagy, the failure of phagophores to mature into autophagic vesicles, or the failure of mature autophagosomes to properly traffic and/or fuse with lysosomes. To define the stage of autophagosome formation that is arrested in *TMEM41B*^*KO*^ cells, we used a series of complementary approaches to systematically probe the autophagosome biogenesis pathway. The accumulation of lipidated LC3 in *TMEM41B*^*KO*^ cells is inconsistent with an initiation defect (Fig 4A). To further support this view, we analyzed the kinase activity of Unc-51 like autophagy activating kinase 1 (ULK1), a component of the RB1CC1 complex that regulates phagophore nucleation [33]. Upon autophagy induction, ULK1 phosphorylates numerous substrates including ATG13. Consequently, the phosphorylation of serine-318 (p-S318) in ATG13 can serve as a proxy for ULK1 activity [34,35]. We found that activation of autophagy by torin stimulated the appearance of p-S318 comparably in wild-type and *TMEM41B*^*KO*^ cells (S4A Fig). For comparison, this assay robustly detected loss of ULK1 activity in cells lacking RB1CC1, a known ULK1 kinase coactivator (S4A Fig). These data argue that the primary role of TMEM41B is downstream of initiation.

**Fig 5:**
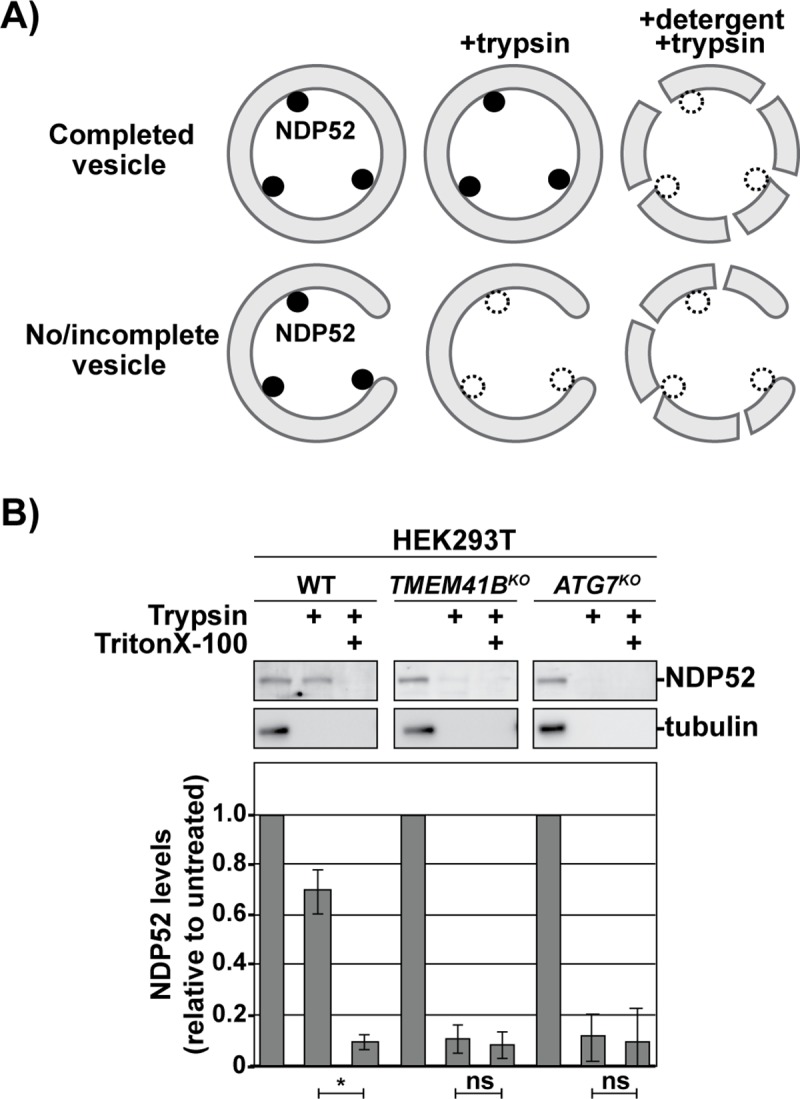
TMEM41B is required for phagophore maturation. (A) Schematic of the protease protection assay for detecting closed autophagosomes. Targets are protected from proteolysis by trypsin when sequestered by properly sealed vesicles. (B) WT and indicated HEK293T knockouts were treated for 18 h with 100 nM BafA1 prior to gentle, mechanical lysis. The corresponding cell extracts were treated as indicated prior to being resolved by SDS-PAGE and analyzed by IB with indicated antibodies. NDP52 serves as a representative autophagy target; tubulin is unincorporated and serves as a control for proteolysis. Bar graphs show the mean ± SD of each sample from 3 independent experiments; *p*-values were determined using a student *t* test. *p < 0.01. Related to S4 Fig. Underlying data for all summary statistics can be found in S1 Data. BafA1, Bafilomycin A1; HEK, human embryonic kidney; IB, immunoblotting; NDP52, nuclear dot protein 52; ns, not significant; TMEM41B, transmembrane protein 41B; WT, wild type.

Next, to determine whether initiated phagophores matured properly in *TMEM41B*^*KO*^ cells, we turned to a protease protection assay (Fig 5A) [36]. In this assay, properly sealed autophagosomes protect their cargo from proteolysis by an exogenous protease. Cells were treated with BafA1 for 18 h to accumulate autophagosomes prior to lysis by mechanical disruption. As expected, in wild-type extracts, the autophagy receptor NDP52 was protease resistant until membranes were solubilized with Triton X-100 (a nonionic detergent). By contrast, NDP52 was protease sensitive in native extracts derived from *TMEM41B*^*KO*^ cells and *RB1CC1*^*KO*^ control cells, consistent with a failure to properly form completed autophagosomes (Fig 5B). We obtained similar results when we analyzed protease protection of LC3-II (S4B and S4C Fig). In sum, these data suggest that TMEM41B is required for phagophore maturation.

**Fig 6:**
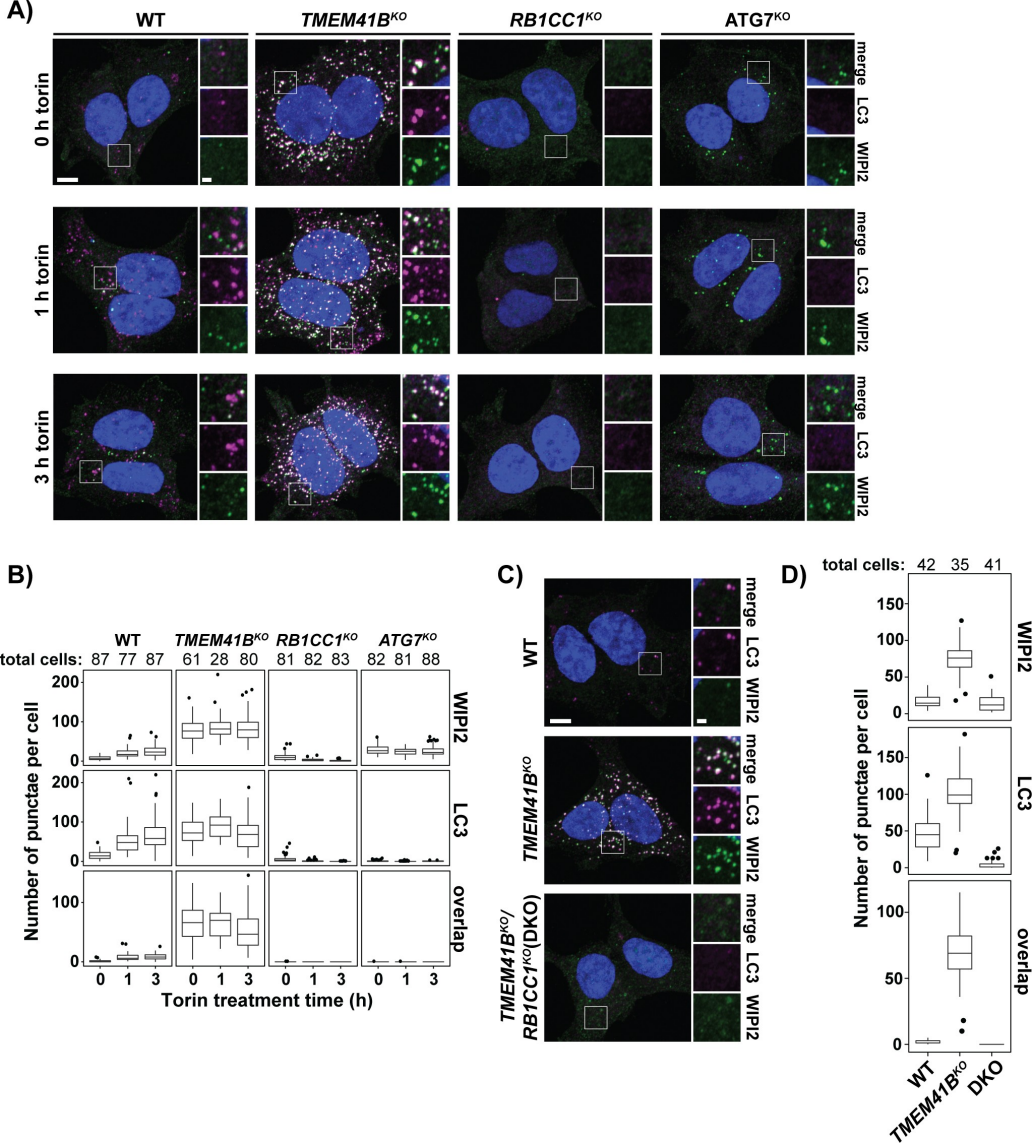
*TMEM41B*^*KO*^ cells accumulate unresolved intermediates in autophagosome biogenesis. (A) Wild-type and indicated HEK293T knockout cells were treated with 250 nM torin for the indicated times prior to analysis by confocal microscopy. Shown are representative confocal micrographs (as maximum intensity projections). Selected regions (white boxes) of micrographs are shown as insets of single and merged channels from IF against indicated proteins. LC3, magenta; WIPI2, green; merged, white; Hoechst, blue. Scale bars: large panels, 5 μm; small panels, 1 μm. (B) Plots showing means of indicated punctae in wild-type and HEK293T knockout cells imaged in part A with inner quartiles (boxed regions), 1.5 interquartile ranges (whiskers), and outliers (dots) indicated. Sample size (*n*) for each sample is indicated. (C) Representative confocal micrographs (as maximum intensity projections) of wild-type and indicated single and DKOs of HEK293T cells analyzed by confocal microscopy. Selected regions (white box) of micrographs are shown as insets of single and merged channels from IF against indicated proteins. LC3, magenta; WIPI2, green; merged, white; Hoechst, blue. Scale bars: large panels, 5 μm; small panels, 1 μm. (D) Plots showing means of indicated punctae in cells imaged in part C with inner quartiles (boxed regions), 1.5 interquartile ranges (whiskers), and outliers (dots) indicated. Sample size (*n*) for each sample is indicated. Related to S5 Fig. Underlying data for all summary statistics can be found in S1 Data. ATG, autophagy-related; DKO, double knockout; HEK, human embryonic kidney; IF, immunofluorescence; LC3, microtubule-associated protein 1 light chain 3B; RB1CC1, RB1 Inducible Coiled-Coil 1; TMEM41B, transmembrane protein 41B; WIPI2, WD Repeat Domain, Phosphoinositide Interacting 2.

To better define the arrested stage of autophagosome biogenesis in *TMEM41B*^*KO*^ cells, we used confocal microscopy to monitor the recruitment of various ATGs associated with specific steps in autophagosome biogenesis. To validate this approach, we monitored the formation of LC3-positive (LC3+) punctae. Consistent with our earlier biochemical approaches (Fig 4, Fig 5; S3 Fig, S4 Fig), we found that *TMEM41B*^*KO*^ cells displayed approximately 5.5-fold more LC3+ punctae than wild-type cells under basal conditions (Fig 6A and 6B). We reached a similar conclusion when we measured the abundance of LC3+ structures that colocalized with SQSTM1 as an additional marker of autophagosome biogenesis (S5A and S5B Fig). Furthermore, torin treatment induced an approximately 4-fold increase in the level of LC3+ punctae in wild-type cells while having no effect on *TMEM41B*^*KO*^ cells (Fig 6A and 6B). These data are broadly consistent with an inhibition of autophagic flux in *TMEM41B*^*KO*^ cells.

We next analyzed LC3+ structures for WIPI2, which associates with PI3P-rich phagophore intermediates but not mature autophagosomes [17,37]. We found that the majority of LC3+ structures in *TMEM41B*^*KO*^ cells colocalized with WIPI2 (79%; *n* = 5,032 LC3+ structures) while those in wild-type cells overlapped poorly with WIPI2 (8%; *n* = 1,394) (Fig 6A and 6B). Genetic ablation of RB1CC1 abolished accumulation of LC3+ or WIPI2+ structures in *TMEM41B*^*KO*^ cells, arguing that these structures represent bona fide intermediates in the process of phagophore maturation (Fig 6C and 6D; S5C Fig). In addition, we visualized syntaxin 17 (STX17), a SNARE protein that is recruited to late phagophore intermediates prior to vesicle closure [38]. This analysis did not find appreciable amounts of STX17 on the LC3+ structures that accumulate in *TMEM41B*^*KO*^ cells (S5D and S5E Fig). For comparison, we recapitulated the published observation that STX17+ structures accumulate in the absence of ATG7 (S5D and S5E Fig) [20].

**Fig 7:**
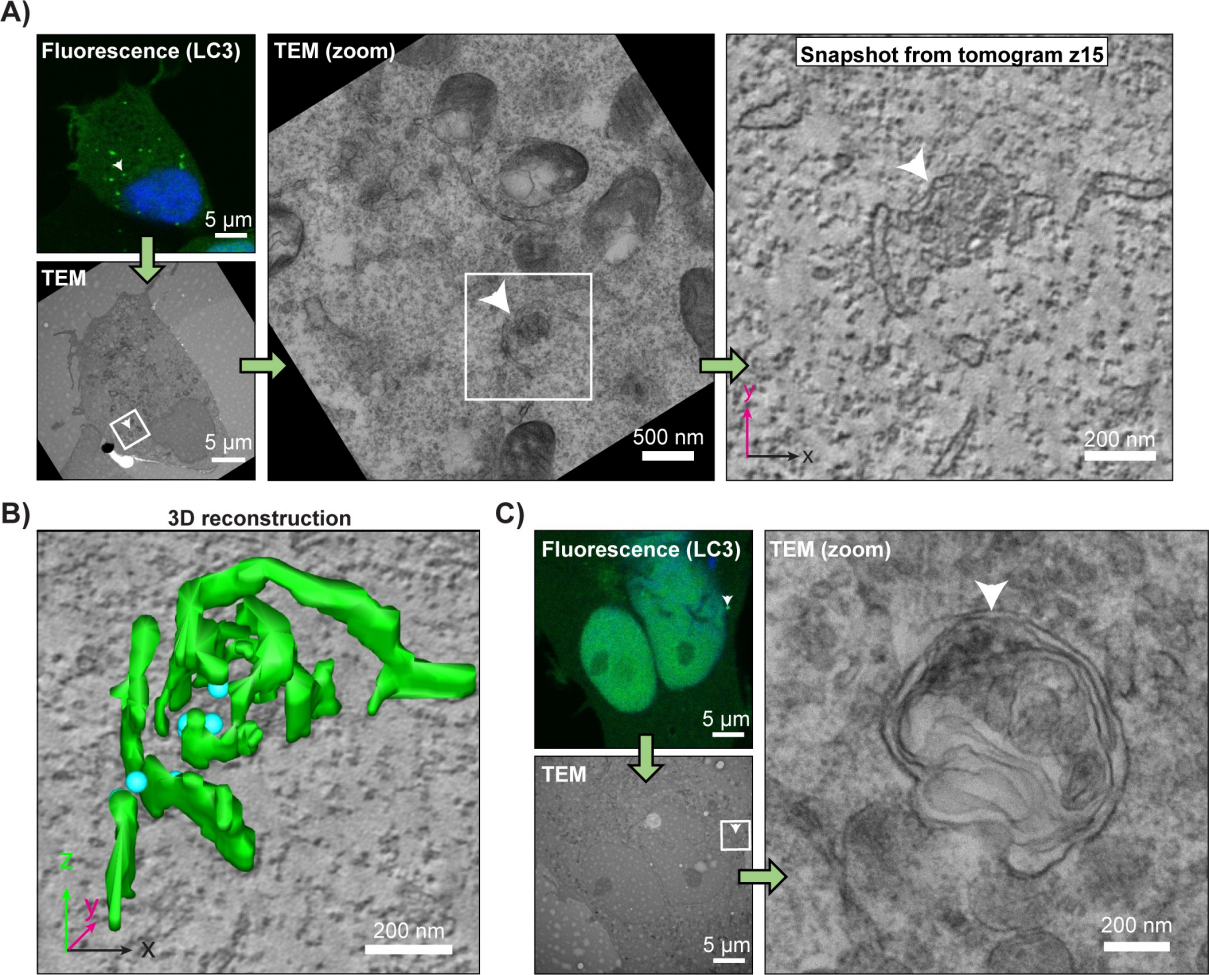
Pre-autophagosomal membranes fail to coalesce in *TMEM41B*^*KO*^ cells. CLEM of HEK293T *TMEM41B*^*KO*^ (A and B) and wild-type HEK293T cells (C) expressing tfLC3 that were grown under basal conditions (no drug treatment). Analysis workflow is indicated by green arrows. White arrowheads indicate a representative structure of interest. White boxes demarcate zoomed areas in subsequent images. Blue indicates Hoechst 33342. Panel A includes a sample tomogram used to make the three-dimensional reconstruction (in B) of the membranous structures found in the GFP-positive region. Green indicates membrane sheets and tubes; cyan indicates membrane vesicles. See Materials and methods for further CLEM analysis details. Related to S6 Fig. CLEM, correlative light and electron microscopy; GFP, green fluorescent protein; HEK, human embryonic kidney; LC3, microtubule-associated protein 1 light chain 3B; TEM, transmission electron microscopy; tf, tandem-fluorescent.

Collectively, our biochemical and microscopy-based data argue that ablation of TMEM41B arrests phagophore maturation during membrane elongation—specifically, after recruitment of LC3 or WIPI2 but prior to WIPI2 dissociation or STX17 recruitment. To substantiate this interpretation, we used transmission electron microscopy (TEM) as an alternative method of visualizing arrested autophagosomal intermediates at an ultrastructural level. Analysis by TEM revealed the accumulation of single-membrane vesicles (approximately 140 nm in diameter) in *TMEM41B*^*KO*^ cells but not in wild-type cells (S6A Fig). However, correlative light and electron microscopy (CLEM) demonstrated that fluorescent LC3 punctae did not correlate with these vesicles but rather with a distinct population of smaller (approximately 50 nm) vesicles (Fig 7A). Three-dimensional reconstruction of electron miscroscopy tomographs containing LC3+ structures revealed a rudimentary collection of membranous sheets, tubules, and vesicles, reminiscent of immature isolation membranes [15] (Fig 7B; S6B and S6C Fig). For comparison, in wild-type cells, LC3 punctae correlated with structures resembling fully-fledged autophagosomes (Fig 7C).

### TMEM41B is functionally related to VMP1

**Fig 8:**
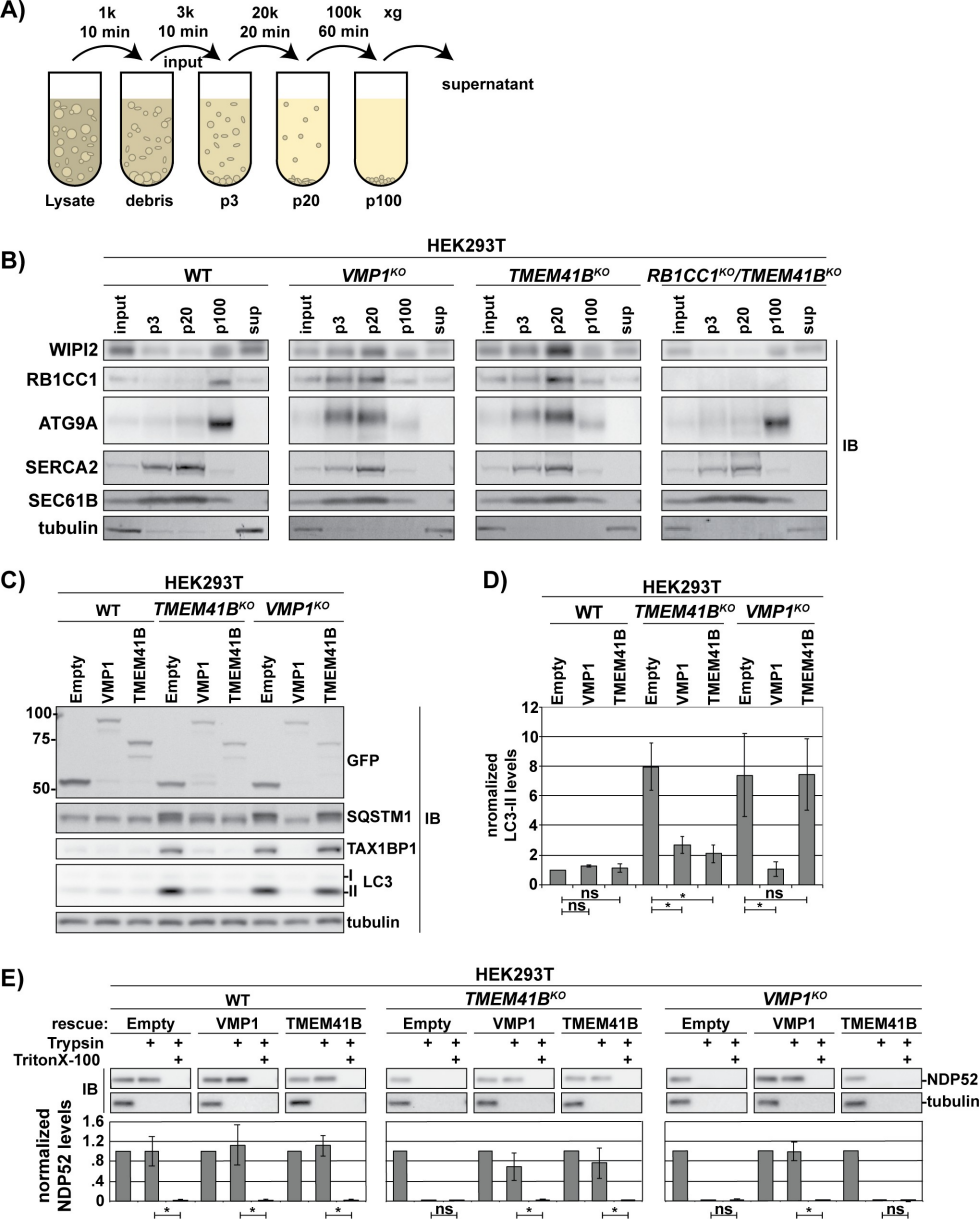
TMEM41B shares structural homology and functional redundancy with VMP1. (A) Schematic for vesicle fractionation protocol. (B) WT and indicated HEK293T knockout cells were fractionated as outlined in panel A. Fractions were resolved by SDS-PAGE followed by IB with indicated antibodies. Membrane fractions are loaded as 15X concentrate. (C) tfEmpty, tfVMP1, or tfTMEM41B expression constructs were integrated at the AAVS1 locus in WT and HEK293T knockout cells. Cell lysates were normalized by total protein prior to resolution by SDS-PAGE and IB with indicated antibodies. LC3-II levels relative to WT (tfEmpty) cells are indicated under each lane (normalized to tubulin). (D) Quantitation of LC3-II levels shown in C. LC3-II levels in each lane were normalized to tubulin. LC3-II levels in WT (Empty) cells were set to 1. Bar graphs show the mean ± SD of each sample from 3 independent experiments; *p*-values were determined using a student *t* test. *p < 0.02. (E) HEK293T cells from C were treated for 18 h with 100 nM BafA1 prior to gentle, mechanical lysis. The corresponding cell extracts were treated as indicated prior to being resolved by SDS-PAGE and analyzed by IB with indicated antibodies. NDP52 serves as a representative autophagy target; tubulin is unincorporated and serves as a control for proteolysis. Bar graphs show the mean ± SD of each sample from 3 independent experiments; *p*-values were determined using a student *t* test. *p < 0.02. Related to S7 Fig. Underlying data for all summary statistics can be found in S1 Data. AAVS1, adeno-associated virus integration site 1; ATG, autophagy-related; BafA1, Bafilomycin A1; GFP, green fluorescent protein; HEK, human embryonic kidney; IB, immunoblotting; LC3, microtubule-associated protein 1 light chain 3B; NDP52, nuclear dot protein 52; ns, not significant; RB1CC1, RB1 Inducible Coiled-Coil 1; SQSTM1, sequestosome 1; Sup, supernatant; TAX1BP1, tax1 binding protein 1; tf, tandem-fluorescent; TMEM41B, transmembrane protein 41B; VMP1, vacuole membrane protein 1; WIPI2,; WT, wild-type.

To gain insight into the biochemical function of TMEM41B, we focused on its broadly conserved transmembrane domain (pfam09335) [39] (S3A Fig; S7A Fig). VMP1 is another broadly conserved ER-membrane protein that also contains a pfam09355 domain and is required for an early stage of autophagy [6,7,14,15,40]. On the basis of these similarities, we hypothesized that TMEM41B and VMP1 have related activities. To examine this possibility, we looked for increased association of certain ATG factors with ER membranes in the absence of TMEM41B, a hallmark of arrested autophagosome biogenesis in *VMP1*^*KO*^ cells [41]. Indeed, following differential centrifugation of cell lysates (Fig 8A), we observed a shift in the migration of WIPI2, RB1CC1, and ATG9A from the high-speed pellet (p100) to lower-speed pellets containing ER-derived membranes (p3 and p20) in both *VMP1*^*KO*^ and *TMEM41B*^*KO*^ cells (Fig 8B). Importantly, we could suppress this effect on WIPI2 and ATG9A fractionation in *TMEM41B*^*KO*^ cells by genetically ablating RB1CC1, highlighting that this is an autophagy-dependent effect.

To additionally test the hypothesis that TMEM41B and VMP1 have related activities, we performed a genetic complementation analysis. Specifically, we used our tf gene cassette system to overexpress tagged versions of TMEM41B and VMP1 and evaluated suppression of the *TMEM41B*^*KO*^ phenotype by 2 assays: monitoring receptor accumulation by IB and cargo encapsulation by protease protection. Both tfTMEM41B and tfVMP1 (approximately 15-fold over-expressed [S7B Fig]) complemented *TMEM41B*^*KO*^ phenotypes (Fig 8C–8E). By contrast, only tfVMP1 was able to suppress *VMP1*^*KO*^ phenotypes. Taken together, these data strongly argue that VMP1 and TMEM41B have partially overlapping roles during phagophore maturation.

## Discussion

The power of forward genetics in yeast is the foundation on which the field of autophagy research firmly stands [1–3,42–46]. Complementary work on autophagy in higher eukaryotes has revealed both the deep conservation of this process, as well as novel mechanisms by which it is regulated in the context of development, immunity, and neuronal homeostasis. Much of this diversification is enabled by autophagy receptors, proteins that connect diverse cellular targets with the core autophagy machinery [47]. However, the potential of these receptors as genetic handles for identifying new factors and adaptations of human autophagy has only begun to be explored.

CRISPR-based screens have recently demonstrated their power over previous technologies in identifying novel mammalian autophagy factors and pathways [48,49]. Here, we combined CRISPR screening with an expanded toolkit of tf reporters to identify novel factors required for mammalian autophagy. These data are freely available in their entirety as an interactive resource at http://crispr.deniclab.com.

Our screens recovered virtually all known ATG factors as well as uncharacterized factors, including TMEM41B. In follow-up studies, we showed that TMEM41B is an ER integral membrane protein critical for autophagic flux in multiple human cell lines. Using complementary biochemical and fluorescence-microscopy–based assays, we detected the accumulation of unsealed, LC3+, WIPI2-positive autophagy intermediates in cells ablated for TMEM41B (Figs 5 and 6). Furthermore, our ultrastructural analysis by CLEM found that these intermediates correspond to a collection of thin (approximately 50 nm) membranous sheets, tubules, and vesicles, which are hallmarks of immature isolation membranes (Fig 7) [15].

TMEM41B contains a conserved transmembrane domain (pfam09335) also found in VMP1, a factor previously known to regulate phagophore maturation [15,41,50]. Correspondingly, overexpression of VMP1 restored autophagosome formation in *TMEM41B*^*KO*^ cells (Fig 8), consistent with the possibility that TMEM41B and VMP1 may be structurally and/or functionally related. VMP1 is absent in yeast but is generally conserved in higher eukaryotes. In *Dictyostelium* and other model eukaryotes, cells lacking VMP1 display diverse phenotypes, including defects in autophagy, membrane contact sites, ion homeostasis, lipid metabolism, and phosphoinositide distribution [6,40,41,50–52]. In light of such pleotropic effects, it remains unclear whether the autophagy defects observed in cells lacking VMP1 or TMEM41B are direct or sequelae of a broader defect in ER-organelle dynamics.

During revision of this manuscript, 2 groups independently reported TMEM41B as a novel modifier of mammalian autophagy [53,54]. Consistent with the results herein, both observed similarities between TMEM41B and VMP1 deletions. On the basis of these phenotypic similarities, one immediate hypothesis is that TMEM41B and VMP1 function as a complex. Indeed, Morita and colleagues were able to isolate a VMP1/TMEM41B-containing co-complex in the presence of n-dodecyl-β-D-maltoside/cholesteryl hemisuccinate (DDM/CHS) [53], although such a complex was not observed in other detergents [54,55]. It remains an important future goal to establish whether TMEM41B’s function is dependent on its interaction with VMP1. A potential clue comes from studies of DedA proteins in *Escherichia coli*, which share pfam09335 with TMEM41B and VMP1 [56] and are distantly related to bacterial LeuT transporters [57]. *E*. *coli* cells lacking the DedA homologs YqjA and YghB have a defect in proton import that can be rescued by overexpressing a Na^+^/K^+^-H^+^ antiporter [58,59]. Future biochemical and structural studies of VMP1 and TMEM41B will provide new tools for testing their potentially conserved role in ion homeostasis.

A deeper understanding of autophagy has the potential to provide therapeutic insight into numerous pathologies, including neurodegenerative disease, cardiometabolic disease, and cancer [60–62]. TMEM41B, for example, has been implicated in diverse pathologies, including spinal muscular atrophy [63], pulmonary carcinoid tumors [64], and coronavirus infection [65]. Our work argues that autophagy should be further examined as a potential etiological factor in these contexts.

The identification of TMEM41B as a novel ATG factor is one facet of this work, but other aspects of our data remain to be explored. These include novel inducers of autophagy, receptor-specific modulators, and additional putative ATG factors. Vacuolar protein sorting 37 homolog A (VPS37A) and VPS25 are 2 factors in the latter category known to assemble into endosomal sorting complexes required for transport (ESCRT). ESCRT components have long been implicated in autophagy and may function at several steps during autophagosome biogenesis, as well as during autophagosome–endolysosomal fusion [66,67]. Many ESCRT components are required for cell division, which complicates analysis of their mutant phenotypes. By contrast, VPS37A is nonessential and offers a potential future handle for further dissecting the mechanistic details of ESCRT’s role in mammalian autophagy.

In sum, the resource presented herein presents 3 concrete advances. First, it illustrates the robust nature of tf flux reporters as genetic screening tools that can be further applied to other receptors and receptor targets. Second, it yields a rich data set (http://crispr.deniclab.com) for further hypothesis testing. And lastly, it provides a new molecular handle (TMEM41B) for further dissecting the enigmatic role of the ER membrane in the process of autophagosome biogenesis.

## Materials and methods

### Antibodies

The following antibodies were used in this study. For IB, all primary antibodies were used 1:1,000 except where otherwise noted; secondary antibodies were used 1:3,000 (HRP) or 1:10,000 (fluorescent). For immunofluorescence (IF), antibody dilutions are noted following each antibody; secondary antibodies were used 1:500. Primary antibodies include the following: mouse anti-SQSTM1 ([1:200 –IF] ab56416, Abcam, Cambridge, United Kingdom), rabbit anti-TAX1BP1 (#5105, Cell Signaling Technology, Danvers, MA), rabbit anti-NDP52 (#9036, Cell Signaling Technology), rabbit anti-LC3B ([1:2,000 –IB] NB100-2220, Novus Biologicals, Centennial, CO), rabbit anti-LC3A/B ([1:100 –IF] #12741S, Cell Signaling Technology), rat anti-tubulin ([1:500 –IB] sc-53030, Santa Cruz, Dallas, TX), anti-p70-S6K (#9202S, Cell Signaling Technology), anti-p70-S6K(pT389) (#9205S, Cell Signaling Technology), rabbit anti-ATG13 (ABC344, MilliporeSigma, Burlington, MA), rabbit anti-ATG13 (p-S318) (NBP2-19127, Novus Biologicals, Centennial, CO), anti-WIPI2 ([1:150 –IF] ab105459, Abcam), rabbit anti-ATG7 (#8558, Cell Signaling Technology), anti-RB1CC1 (#12436S, Cell Signaling Technology, Danvers, MA), mouse anti-GFP (#11814460001, MilliporeSigma), mouse anti-SERCA2 (ab2861, Abcam), and rabbit anti-Calnexin (#2679, Cell Signaling Technology). Secondary antibodies for IB include the following: goat anti-mouse IgG HRP (#170–6516, Bio-Rad, Hercules, CA), goat anti-rabbit IgG HRP (#170–6515, Bio-Rad, Hercules, CA), goat anti-rat IgG Alexa Fluor 488 (A11006, Thermo Fisher Scientific, Waltham, MA), goat anti-mouse IgG Cy5 (A10524, Thermo Fisher Scientific), and goat anti-rabbit IgG Alexa Fluor 546 (A11010, Thermo Fisher Scientific). Secondary antibodies (for IF) include the following: goat anti-rabbit IgG Alexa Fluor 568 (A11036, Thermo Fisher Scientific) and goat anti-mouse IgG Alexa Fluor Plus 488 (A32723, Thermo Fisher Scientific).

### Chemicals and reagents

The following chemicals and reagents were used in this study: torin1 (sc-396760, Santa Cruz, Dallas, TX), BafA1 (tlrl-baf1, InvivoGen, San Diego, CA), poly-l-lysine (P4707-50ML, MilliporeSigma), Lipofectamine 3000 (L3000008, Thermo Fisher Scientific), nucleofector kit T (VACA-1002, Lonza, Basel, Switzerland), polybrene (H9268-5G, MilliporeSigma), normocin (ant-nr-1, InvivoGen), puromycin (ant-pr-1, InvivoGen), blasticidin (ant-bl-1, InvivoGen), zeocin (ant-zn-1, InvivoGen, San Diego, CA), normal goat serum (NGS; ab7481, Abcam, Cambridge, United Kingdom), Phusion High-Fidelity DNA polymerase (M0530L, NEB, Ipswich, MA), T5 exonuclease (M0363S, NEB, Ipswich, MA), and Taq DNA ligase (M0208L, NEB, Ipswich, MA).

### Vectors

pHR-SFFV-GFP1-10 (Addgene plasmid # 80409) and pCDNA CMV mCherry-GFP-11 were gifts from Bo Huang. pMRXIP GFP-Stx17TM was a gift from Noboru Mizushima (Addgene plasmid #45910). Human Brunello CRISPR knockout pooled library was a gift from David Root and John Doench (Addgene #73178). lentiCRISPRv2 puro was a gift from Brett Stringer (Addgene plasmid #98290). lentiGuide-puro was a gift from Feng Zhang (Addgene plasmid #52963). ptfLC3 was a gift from Tamotsu Yoshimori (Addgene plasmid #21074). pX330-U6-Chimeric_BB-CBh-hSpCas9 was a gift from Feng Zhang (Addgene plasmid #42230). AAVS1-CAG-hrGFP was a gift from Su-Chun Zhang (Addgene plasmid #52344). psPAX2 was a gift from Didier Trono (Addgene plasmid #12260). pCMV-VSV-G was a gift from Bob Weinberg (Addgene plasmid #8454). pUMVC was a gift from Bob Weinberg (Addgene plasmid #8449). pFUGW-EFSp-Cas9-P2A-Zeo (pAWp30) was a gift from Timothy Lu (Addgene plasmid #73857).

### Isothermal assembly

PCR fragments were generated using 2X phusion master mix (M0531S, NEB, Ipswich, MA) and insert-specific primers that appended a 30 bp overlap with target DNA. Vector backbones were linearized by restriction enzyme and dephosphorylated by calf intestinal phosphatase (M0290S, NEB, Ipswich, MA). Prior to assembly, all DNA fragments were gel purified (D4002, Zymo Research, Irvine, CA). Linearized vector DNA (50 ng) was combined with isomolar amounts of purified insert(s); 5 ul of the resulting DNA mix was added to isothermal assembly master mix and incubated at 50°C for 20 min [68]. Assembled product was transformed into NEB Stable competent cells (C3040H, NEB, Ipswich, MA) and plated on LB + agar plates (plus appropriate antibiotics) to isolate single isolates. Single isolates were grown in LB broth + antibiotics, and plasmid DNA was purified using a Qiagen miniprep kit (#27106, Qiagen, Hilden, Germany). Sequences were verified by Sanger sequencing (Eton Bioscience, San Diego, CA).

### sgRNA oligonucleotide ligation protocol

sgRNA oligonucleotides (oligos) were ordered from Eton Bioscience (San Diego, CA). Oligo sequences are listed in S1 Table. To generate the necessary overhangs, all oligos were in the form: Forward: 5′- CACCGNNNNNNNNNNNNNNNNNNNN–3′; Reverse: 5′- AAACNNNNNNNNNNNNNNNNNNNNC–3′. Oligos were diluted to 10 uM in distilled water. A total of 50 pmol each of forward and reverse oligo was combined in a 25 ul reaction and phosphorylated with T4 polynucleotide kinase (M0201S, NEB, Ipswich, MA) in 1X T4 DNA ligase buffer (B0202S, NEB, Ipswich, MA) for 30 min at 37°C. Phosphorylated oligos were boiled for 5 min at 95°C and slow cooled (0.1°C/s) to facilitate annealing. Annealed oligos were diluted 1:100, and 2 μl of insert was ligated into 20 ng digested vector (pLentiuGuide-puro, BsmBI site; pLentiCRIPSR version 2, BbsI site; pX330-derivatives, BbsI site) using T4 DNA ligase (M0202S, NEB, Ipswich, MA). Ligation was allowed to proceed for 15 min at room temperature. Ligated products were transformed into NEB Stable cells (NEB, Ipswich, MA).

### T7 endonuclease (surveyor) assay

Genomic DNA (gDNA) was extracted using QuickExtract buffer (QE0905T, Epicentre, Madison, WI) according to the manufacturer’s instructions. gDNA was subsequently normalized to 200 ng/μl. Per 100 μl PCR, 600 ng gDNA was used as template to amplify the targeted region of interest. Primers are listed in S2 Table. The resulting PCR amplicon was purified using a Zymoclean Gel DNA Recovery Kit (D4002, Zymo Research, Irvine, CA) and normalized to 20 ng/μl in 19 μl 1X NEBuffer 2 (B7002S, NEB, Ipswich, MA). Amplicon was boiled and cooled (−1°C/s) to allow for hybridization. T7 endonuclease I (M0302, NEB, Ipswich, MA) was added at 1 μl per 20 μl reaction and incubated for 15 min at 37°C. Reaction was quenched by adding 1.5 μl 0.25 M EDTA and was analyzed in a 2% UltraPure Agarose gel (#16500500, Thermo Fisher Scientific, Waltham, MA).

### Generation of AAVS1-targeting tfReporters

The hrGFP fragment was excised from AAVS1-CAG-hrGFP using SalI/EcoRV and was replaced with RFP-GFP subcloned from ptfLC3 to generate pCS418 tfEmpty (puro). LC3B (NP_074058.2), SQSTM1 (NP_003891.1), NDP52 (NP_005890.2), NBR1 (NP_006015.4), or TAX1BP1 (NP_005822.1) was PCR amplified and subcloned into the KpnI site of pCS418. Primers are listed in S3 Table. To generate blasticidin-resistant versions of each cassette, the puromycin resistance gene was excised from pCS418 with an XhoI/SpeI digest and replaced with an analogous geneblock fragment (Integrated DNA Technologies, Coralville, IA) encoding a blasticidin resistance gene (BSD).

### Tissue culture

All cells were grown in a standard water-jacketed incubator with 5% CO_2_. K562 cells were grown in IMDM media (#30–2005, ATCC, Manassas, VA) with 10% FBS (#30–2020, ATCC, Manassas, VA) and 1x penicillin/streptomycin. Cells were maintained below 1 million cells per milliliter. HEK293T cells were grown in DMEM media (#30–2002, ATCC, Manassas, VA) with 10% FBS and 1x penicillin/streptomycin. Normocin (1:500) was used as a common additive. All cells were passaged less than 25 times. For passaging, cells were trypsinized with Trypsin-EDTA (#25300–054, Thermo Fisher Scientific, Waltham, MA). Puromycin (2 μg/ml), blasticidin (5 μg/ml), and zeocin (50 μg/ml) were added when necessary for selection. Hct116 cells were grown in McCoy’s 5a modified media (#30–2007, ATCC, Manassas, VA) with 10% FBS (#30–2020, ATCC, Manassas, VA) and 1x penicillin/streptomycin.

### Cell line authentication

gDNA was isolated from HEK293T and K562 cells using the GenElute Mammalian Genomic DNA Miniprep Kits (MilliporeSigma, Burlington, MA). Short tandem repeat (STR) profiling and allele identification were performed by the Molecular Diagnostics Laboratory of Dana-Farber Cancer Institute. Briefly, isolated gDNA was analyzed with the GenePrint 10 STR profiling kit (Promega, Madison, WI) and Amelogenin for gender identification. GeneMapper version 4 Fragment Analysis software (Thermo Fisher Scientific, Waltham, MA) and GenePrint10 allele panel (Promega, Madison, WI) custom bin files were used to identify the alleles at 8 STR loci (TH01, TPOX, vWA, CSF1PO, D16S539, D7S820, D13S317, and D5S818). The ATCC STR Profile Database was used to verify that the identified alleles matched those of the expected cell type.

### Transient transfection and nucleofection

Prior to transfection, HEK293T cells were seeded in OptiMEM Reduced Serum media (#51985–034, Thermo Fisher Scientific, Waltham, MA). At 90% confluency, cells were transfected overnight using Lipofectamine 3000 reagent according to the manufacturer’s recommendations. The following morning, media were exchanged, and cells were passaged for another 24 h prior to drug treatment. K562 cells were nucleofected using a Nucleofector 2b Device (Lonza, Basel, Switzerland) using nucleofector kit T (VACA-1002, Lonza, Basel, Switzerland) and protocol T-016. Transfected/nucleofected DNA was prepared using a ZymoPURE Plasmid Midiprep Kit (D4200, Zymo Research, Irvine, CA).

### Generation of gene knockout cell lines using CRISPR-Cas9 gene editing

For the generation of stable knockouts, HEK293T and K562 cells were transfected or nucleofected, respectively, as described above. Oligo sequences for sgRNAs were generated by CHOPCHOP or were extracted from the Brunello library and cloned into the indicated vectors as outlined above under “sgRNA oligonucleotide ligation protocol” [25,69]. Oligos are listed under S2 Table. Cells were diluted by limiting dilution or cell sorting into 96-well plates and clonally expanded. Expanded cells were confirmed for knockout by western blot or PCR/T7 endonuclease testing.

### Generation of GFP1–10 cell line

HEK293T cells were transduced with a lentiviral GFP1–10 expression cassette. Clonal cell lines were established by limiting dilution and were validated for GFP1–10 insertion by transient transfection with mCherry-GFP-11. Four successful clones were saved, and 1 was used for all further experiments. To tag TMEM41B, cells were transfected with pCS651 (co-expressing Cas9-T2A-BFP, sgTMEM41B) and oVD6217 (an oligo containing 5′ homology arm-GFP11-linker-3′ homology arm). Successful integrants were identified and sorted by FACS.

### AAVS1 integration

Cell lines were co-transfected with pX330-U6-Chimeric_BB-CBh-hSpCas9-AAVS1- gRNA and AAVS1-tfReporter template vectors. Forty-eight h post transfection, cells were incubated with appropriate selection media and passaged for 14 d. Red+/Green+ cells were sorted and propagated.

### Lentiviral generation

Lentivirus was generated in HEK293T cells using Lipofectamine 3000. Cells were grown overnight in OptiMEM media (5% FBS, no antibiotics) (#31985062, Thermo Fisher Scientific, Waltham, MA) to 90% confluency. Cells were than transfected with pVSV-G, pSPAX2, and packaging constructs at a 1:3:4 ratio. Transfection proceeded for 6 to 8 h before media were refreshed. Virus was collected and pooled at 24 and 48 h post transfection. Virus was pelleted at 1,000 g 2× 10 min, aliquoted, and frozen in single-use aliquots. For retrovirus production, all methods were the same except that pUMVC was exchanged for pSPAX2.

### Viral transduction

Cells were incubated in appropriate media containing 8 μg/ml polybrene and lacking penicillin/streptomycin. Cells were transduced overnight. Media were exchanged for media lacking polybrene for 24 h prior to antibiotic selection.

### Protease protection assay

Our protocol for mammalian protease protection assay was based on Zhao and colleagues [36]. Cells were seeded so they would be 75% confluent at 5 PM. Media were then exchanged into media containing 100 nM BafA1. Cells were incubated for 15 h. After incubation, cells were trypsinized and pelleted. Cells were washed 1 time with cold PBS and resuspended in prechilled lysis buffer (20 mM Hepes KOH [pH 7.4], 0.22 M mannitol, 0.07 M sucrose). Cells were lysed by extrusion through a 26-gauge needle 20 times. Samples were pelleted 2 times at 500 g for 10 min at 4°C to pellet debris. When indicated, samples were incubated with 1X trypsin (T1426-100MG [MilliporeSigma, Burlington, MA]; 100X stock: 1 mg/ml) and/or 0.5% Triton X-100 for 35 min at 30°C. Reactions were quenched in 1X hot Laemmli sample buffer and held at 65°C for 10 min.

### Differential centrifugation

Cells were harvested from two to four 15-cm dishes of cells at 95% confluency by scraping and washing them with 5 mL of cold PBS per plate in the cold room. Cells were pelleted at 150 g for 10 min at 4°C. PBS was removed, and cells were resuspended in 3X volume of hypotonic lysis buffer (10 mM HEPES, 10 mM KOAc, 1.5 mM Mg(OAc)_2_, 1X protease inhibitor tablet). Cells were incubated on ice for 20 min and then pelleted. Pelleted cells were resuspended in Buffer E (2X volume of packed cell pellet; 20 mM HEPES [pH 7.4], 250 mM sucrose, 1 mM EDTA, 1X protease inhibitor tablet). Cells were mechanically lysed through a 26-gauge needle using a 1 mL syringe (prechilled at −20°C) on ice (up/down 8 times, letting cells settle in between). Lysate was diluted 2X in Buffer E prior to pelleting. Lysate was cleared cell debris and nuclei by pelleting at 1,000 *g* for 10 min at 4°C. Sequential pelleting was performed at 3,000 *g* for 10 min, 20,000 *g* for 20 min, and 100,000 *g* for 60 min. All pellets were resuspended in Buffer E.

### Immunoprecipitation

Cells were collected and resuspended in IP buffer (50 mM HEPES [pH 7.4], 150 mM NaCl, 2 mM EDTA, 1% Triton X-100). Cells were incubated on ice for 30 min and pelleted twice at 5,000 g for 5 min at 4°C. Supernatant was applied to prewashed GFP-Trap or RFP-Trap magnetic agarose (Chromotek, Planegg-Martinsried, Germany) and incubated for 1 h at 4°C. Beads were washed 4× 5 min with 2 tube changes. Protein was eluded by boiling at 70°C in 1X SDS buffer.

### Gel electrophoresis and western blotting

Cells were trypsinized at 75% confluency and quenched in an equal amount of media. Cells were lysed for 15 min on ice in lysis buffer (50 mM HEPES [pH 7.4], 150 mM NaCl, 2 mM EDTA, 1% Triton X-100, 2X complete protease inhibitor tablet [Roche, Basel, Switzerland]). For phosphorylation analysis, lysis buffer was supplemented with phosphatase inhibitors (10X: 100 mM NaF, 10 mM Na_3_VO_4_, 100 mM NaPP_i_). Lysates were cleared 2X at 1,000 g for 5 min. Post-spin supernatants were used as input. Protein levels in supernatants were normalized using a BCA protein assay (#23227, Thermo Fisher Scientific, Waltham, MA). Normalized samples were boiled in 1X (final concentration) Laemmli Loading Buffer (3X stock: 189 mM Tris [pH 6.8], 30% glycerol, 6% SDS, 10% beta-mercaptoethanol, bromophenol blue). Gel electrophoresis was performed at 195 V for 70 min in Novex 4%–20% Tris-Glycine gels. Total protein analysis was performed using SYPRO Ruby (Thermo Fisher Scientific, Waltham, MA) according to the manufacturer’s recommendations (short protocol). For western blotting, samples were transferred for 60 min to 0.2 μm PVDF membranes (#ISEQ00010, MilliporeSigma, Burlington, MA) using a Semi-dry transfer cell (Bio-Rad, Hercules, CA). Membranes were blocked for 20 min in TBS-T with 5% milk. Primary antibodies were incubated overnight at 4°C. Blots were then rinsed 3× 5 min. Secondary antibodies were incubated for 1 h at room temperature. Blots were rinsed 4× 10 min in TBS-T and imaged using fluorescence (Typhoon Trio Imager, GE Healthcare, Chicago, IL) or chemiluminescence (SuperSignal West Femto Maximum Sensitivity Substrate, #34095, Thermo Fisher Scientific, Waltham, MA). If necessary, stripping of membranes was performed using Restore Western Blot Stripping Buffer (#21059, Thermo Fisher Scientific, Waltham, MA) for 10 min.

### IF and immunocytochemistry

Coverslips (#12-548A, Thermo Fisher Scientific, Waltham, MA) were placed in 6-well tissue culture plates (#62406–161, VWR, Radnor, PA) and coated with poly-L-lysine (#P4707, MilliporeSigma, Burlington, MA) per the manufacturer’s recommendations. Cells were seeded onto coverslips overnight so that they would be 15% confluent at the time of fixation. When reported, cells were treated with 250 nM torin for 1 to 3 h prior to fixation. Fresh 16% PFA (#15710, Electron Microscopy Sciences, Hatfield, PA) was diluted to 4% in 1X Dulbecco’s phosphate buffered saline with calcium chloride and magnesium chloride (#14080–055, Thermo Fisher Scientific, Waltham, MA). Coverslips were removed with forceps and placed into 4% PFA for 15 min. PFA was aspirated and washed twice with PBS (D8537, MilliporeSigma, Burlington, MA). For LC3 IF, cells were transferred to wells containing prechilled (−20°C) methanol for 5 min. Slides were returned to PBS and washed 2× for 5 min. Slides were blocked at RT for 1 h in blocking buffer (0.3% Triton X-100, 5% NGS in PBS) and washed once in PBS. Primary antibody was diluted in 5% NGS at the dilutions described elsewhere in Materials and methods. The amount of 75 ul of antibody mixture was spotted on parafilm in a humidified chamber, and inverted coverslips were incubated with antibody overnight at 4°C. After incubation, coverslips were washed 3× 10 min in PBS. Secondary antibodies were diluted 1:500 in 5% NGS, and inverted coverslips were incubated with antibody mixture for 45 min. Cells were stained with a 1:10,000 dilution of Hoechst 33342 (H3570, Thermo Fisher Scientific, Waltham, MA) for 5 min. Coverslips were washed 4× 10 min in PBS and mounted on coverslips (#294875X25, Corning, Corning, NY) using Prolong Diamond (P36965, Thermo Fisher Scientific, Waltham, MA).

### Confocal microscopy

Fluorescent images were obtained using a confocal microscope with Airyscan detectors (LSM880 with Airyscan, Zeiss) and a 63X PlanAPO oil-immersion objective lens (Zeiss, Oberkochen, Germany) and were processed with Zeiss Blue software (Zeiss, Oberkochen, Germany).

### Image analysis

Fluorescence microscopy images were processed using a newly developed Python analysis pipeline built around the pyto_segmenter analysis package [70]. First, regions of images containing cells were identified. To do so, we first fit a Gaussian distribution to the fluorescence intensity distribution for a smoothed green channel (488 nm excitation) z-stack from an empty field. Using this Gaussian fit, we predicted the probability that each pixel in the smoothed green channel z-stack for each field corresponded to background (noncell) or foreground (cell). We assigned each pixel with a *p*(background) < 10^−5^ to the cells, thus creating a “cell mask.” After removing small specks (<100,000 pixels volume) to eliminate debris, we removed out-of-focus planes from the cell mask using a Support Vector Machine (SVM) classifier as described previously [71]. Next, nuclei were segmented from the blue (DAPI) channel by slice-by-slice relative thresholding followed by watershed segmentation using the pyto_segmenter package. Cells were segmented using watershed segmentation from nuclei seeds. Cell edges were eroded to eliminate blurred edge excess included during the *p*-value transformation. Cells contacting the edge of the field were removed from analysis. Next, punctae were segmented in the green and red (561 nm) channels using the pyto_segmenter package with empirically determined Canny edge detection thresholds. The number of total punctae and punctae overlapping with objects in the other fluorescence channel were counted, and tabulated data were saved in .csv format. Plotting was performed using R and the ggplot2 package. See the image analysis package for details. Scripts for image analysis and data plotting can be found at https://github.com/deniclab/csth-imaging/tree/pub_version.

### Sample size estimate and experimental replication details

For microscopy experiments, 40 images were collected, and the number of cells in each sample was counted using segmentation scripts. Cell counts are indicated above each quantitation. Samples were masked prior to data collection and analyzed using automated scripts to eliminate bias during quantification. Replicates represent biological replicates in which strains were subjected to identical preparations on different days.

For sequencing experiments, the number of replicates (2–4) are indicted in S2 Data. Each replicate was a biological replicate in which strains were transfected and taken through the entire experiment on separate days.

For flow cytometry experiments, *n* is indicated for each experiment in the figure legend; >1,000 cells were used for each experiment.

### Library propagation

Brunello library (2-vector system) was purchased from addgene (item #73178). The amount of 50 ng of library was electroporated into 25 μl Endura electrocompetent cells (60242–2, Lucigen, Middleton, WI). Cells from 8 electroporations were pooled and rescued in 8 ml of rescue media for 1 h at 37°C. Eight milliliters of SOC (2% tryptone, 0.5% yeast extract, 10 mM NaCl, 2.5 mM KCl, 10 mM MgCl_2_, 10 mM MgSO_4_, and 20 mM glucose) was added to cells, and 200 μl of the final solution was spread onto 10 cm LB plates containing 50 μg/ml carbenicillin (80 plates total). Through a dilution series, 500 million colonies were estimated, representing 7,000X coverage of the library. Cells were manually scraped off plates, and a GenElute Megaprep kit (NA0600-1KT, MilliporeSigma, Burlington, MA) was used to purify plasmid DNA.

### Library lentiviral generation

Lentivirus was generated by lipofection (Lipofectamine 3000) of HEK293T cells with 5 μg psPAX2 (Addgene Plasmid #12260), 1.33 μg pCMV-VSV-G (Addgene plasmid Plasmid #8454), and 4 μg library vector per 10 cm plate. Transfection was performed according to the manufacturer’s specifications. Briefly, low-passage HEK293T cells were grown in OptiMEM + 5% FBS medium to 95% confluency by time of transfection. Cells were transfected for 6 h, and then media were replaced with fresh OptiMEM + 5% FBS. At 24 h post transfection, supernatant was collected and replaced. At 48 h post transfection, supernatant was again collected, pooled with the 24 h supernatant, and clarified 2× 1,000 g for 10 min. Viral RNA was purified using a Macherey Nagel viral RNA purification kit (Macherey-Nagel, Düren, Germany). Viral RNA was quantified using the Lenti-X qRT-PCR Titration Kit (Clontech, Mountain View, CA). A value of 849 copies/IFU, derived from a control virus expressing BFP, was used to calculate viral titer.

### Transduction and cell growth

For CRISPR screening experiments, K562 cells were passaged to maintain cell density between 500,000 and 2 million cells/ml. Cells were propagated in IMDM + 10% FBS + penicillin/streptomycin + appropriate antibiotics (blasticidin 5 μg/ml, zeocin 50 μg/ml) until 200 million cells were obtained (approximately 8–10 d). For infection, 200 million cells were pelleted and resuspended in IMDM + 10% FBS + 8 μg/ml polybrene. Date of infection was day 0. An MOI of 0.4 was used to minimize multiple infection events per cell. Cells were infected overnight, pelleted, and exchanged into fresh media. After 24 h, cells were split, and 2 μg/ml puromycin was added. Cells were continually passaged in puromycin. At day 10, cells were removed from puromycin, and at day 12, cells were sorted for Red:Green fluorescence. The amount of 100 M unsorted cells were pelleted and processed as input. The top and bottom 30% of cells (based on Red:Green ratio) were taken; 100 million cells were sorted for each experimental condition. Cell sorting was performed using a FACSAria (Becton Dickinson, Franklin Lakes, New Jersey) or BioRad S3 (Bio-Rad, Hercules, CA) sorter. Cells were pelleted and stored at −80°C until processing.

### CRISPR screen processing

gDNA was purified from collected cells using the NucleoSpin Blood XL kit (#740950.1, Macherey-Nagel, Düren, Germany) according to the manufacturer’s instructions. Illumina sequencing libraries were created by PCR amplifying the genomically integrated sgRNA sequences. All gDNA was used for each PCR. A pool of 8 staggered-length forward primers was used in each PCR reaction to avoid monotemplating during Illumina sequencing. Reverse primers contained unique barcodes designed to allow for sequencing and differentiation of multiple libraries on a single chip during Illumina sequencing. Each 50 μL PCR reaction contained 0.4 μM of each forward and reverse primer mix (Integrated DNA Technologies), 1X Phusion HF Reaction Buffer (NEB, Ipswich, MA), 0.2 mM dNTPs (NEB, Ipswich, MA), 40 U/mL Phusion HF DNA Polymerase (NEB, Ipswich, MA), 5 μg of gDNA, and 3% v/v DMSO. The following PCR cycling conditions were used: 1X 98°C for 30 s; 25X (98°C for 30 s, 56°C for 30 s, and 72°C for 30 s); and 1X 72°C for 10 min. The resulting products were pooled to obtain the sgRNA libraries. The pooled PCR products were size selected by adding 0.95X magnetic bead slurry as outlined by DeAngelis and colleagues [72]. The High Sensitivity D1000 ScreenTape system (Agilent Technologies, Santa Clara, CA) was used to confirm the absence of primer dimers after purification. Sample libraries were quantified by qPCR using the NEBNext Library Quant Kit for Illumina (NEB, Ipswich, MA). Four to five libraries were pooled to a total concentration of 10 nM for simultaneous Illumina sequencing on a single chip. The libraries were sequenced using either the HiSeq 2000 or 2500 system (Illumina). The HiSeq 2000 system was run on High Output Run Mode with the TruSeq SBS version 3 kit (Illumina, San Diego, CA). The HiSeq 2500 system was run on either the Rapid Run Mode with the HiSeq Rapid SBS version 2 kit (Illumina, San Diego, CA) or the High Output Run Mode with the HiSeq SBS version 4 kit (Illumina, San Diego, CA). Sequencing was performed per recommendations of the manufacturer with custom sequencing and indexing primers (Integrated DNA Technologies, Coralville, IA). For primer sequences, see S4 Table.

### NGS data analysis

The 5′ end of NGS reads were trimmed to 5′-CACCG-3′ using Cutadapt. The count function of MAGeCK (version 0.5.3) was used to extract read counts for each sgRNA. Raw read counts can be found in S2 Data. The mle function was used to compare read counts from cells displaying increased and decreased Red:Green ratios. The output included both beta scores and false discovery rates. All beta scores can be found in S3 Data. Beta scores for each sgRNA for each tfReporter were averaged across 2 to 4 experiments. Across all experiments, average read counts were 200 to 400 per sgRNA. To generate heat maps for each reporter (e.g., Fig 2), the beta scores for each gene were normalized by the beta score for ATG9A.

### Flow cytometry

All samples were pelleted, washed 1× in cold PBS, and filtered through strainer cap tubes (21008–948, VWR, Radnor, PA) prior to analysis. Flow cytometry data were collected on an LSRII flow cytometer. Data were analyzed in FlowJo (FlowJo LLC, Ashland, Oregon) and R. The biomodality of populations was determined in an unbiased manner using the BifurGate tool in FlowJo. At least 1,000 cells were collected for all samples.

### CLEM

For CLEM, HEK293T *TMEM41B*^KO^ or wild-type cells stably expressing GFP-mCherry-LC3 were grown on photo-etched coverslips (Electron Microscopy Sciences, Hatfield, PA). Cells were fixed in 4% formaldehyde, 0.1% glutaraldehyde/0.1 M PHEM (240 mM PIPES, 100 mM HEPES, 8 mM MgCl_2_, 40 mM EGTA [pH 6.9]) for 1 h. The coverslips were washed with 0.1 M PHEM buffer and mounted with Mowiol containing 1 μg/ml Hoechst 33342. The cells were examined with a Zeiss LSM780 confocal microscope (Carl Zeiss MicroImaging GmbH, Jena, Germany) utilizing a Laser diode 405–30 CW (405 nm) and an Ar-Laser Multiline (488 nm). Cells of interest were identified by fluorescence microscopy; a z-stack covering the whole cell volume (voxel size: 83 nm × 83 nm × 438 nm) was acquired. The relative positioning of the cells on the photo-etched coverslips was determined by taking a low magnification DIC image. The coverslips were removed from the object glass, washed with 0.1 M PHEM buffer, and fixed in 2% glutaraldehyde/0.1 M PHEM overnight. Cells were postfixed in osmium tetroxide and potassium ferry cyanide, stained with tannic acid and uranyl acetate, and thereafter dehydrated stepwise to 100% ethanol followed by flat-embedding in Epon. Serial sections (200 nm) were cut on a Ultracut UCT ultramicrotome (Leica, Wetzlar, Germany) and collected on formvar-coated slot-grids.

Sections were observed at 200 kV in a Thermo Scientific Talos F200C microscope and recorded with a Ceta 16M camera. Consecutive sections were used to align electron micrographs with fluorescent images in X, Y, and Z. For tomograms, image series were taken between −60° and 60° tilt angles with 2° increment. Single-tilt axes series were recorded with a Ceta 16M camera. Tomograms were computed using weighted back projection using the IMOD package. Display, segmentation, and animation of tomograms were also performed using IMOD software version 4.9 [73].

## Supporting information

S1 Fig (Related to Fig 1) Generation and validation of tfReceptor autophagy reporters. (A) Diagram of gene cassette used for expressing all tf proteins in this study. Salient cassette features are color coded. Shown below are ATG factors that were expressed as N-terminal tf fusions with their length, common names, and RefSeq accession number listed left to right. All inserts were positioned at the KpnI restriction site. (B) HEK293T cells expressing indicated tf expression cassettes from the AAVS1 locus were analyzed by flow cytometry under basal conditions and after 18 h treatment with 250 nM torin. Plots show median Red:Green ratios, inner quartiles (boxed regions), and 10th and 90th percentile (whiskers). All samples are normalized to basal Red:Green ratio. (C) HEK293T cells expressing the indicated tf proteins from the AAVS locus were grown under basal conditions or treated with torin. Shown are flow cytometry traces of GFP and RFP fluorescence (arbitrary units), both as individual signals and as a ratio (Red:Green). (D) Extracts derived from cells with indicated genotypes were normalized by total protein levels using a BCA assay and resolved by SDS-PAGE followed by IB with indicated antibodies. (E) Wild-type and indicated HEK293T knockout cells expressing tfSQSTM1 from the AAVS1 locus were treated and analyzed as in part B. Underlying data for all summary statistics can be found in S1 Data. AAVS1, AAVS homology arms; ATG, autophagy-related; BGH pA, bovine growth hormone polyadenylation signal; CAG, CAG promoter sequence; GFP, green fluorescent protein; IB, immunoblotting; P2A, self-cleaving peptide; Puro^R^/BSD^R^, puromycin or blasticidin resistance cassette; RFP, red fluorescent protein; SA, splice acceptor; tf, tandem-fluorescent.
(TIF)

S2 Fig (Related to Fig 3) Confirmation of effects of novel autophagy factors. (A–D) K562 cells co-expressing Cas9 and indicated tfReporters were transduced with individual sgRNAs against the shown genes or with nontargeting sgRNA controls. Cells were treated and analyzed as in Fig 3A. These data are represented as part of the heat map in Fig 3B. *n* > 5,000 cells each. (E) K562 cells co-expressing Cas9 and tfLC3 were transduced with sgRNAs against the indicated genes or with a negative sgRNA control. Shown are flow cytometry traces of GFP and RFP fluorescence (in arbitrary units), both as individual signals and as a ratio (Red:Green). Cells were treated and analyzed as in panel A. Underlying data for all summary statistics can be found in S1 Data. Cas9, CRISPR-associated protein 9; GFP, green fluorescent protein; RFP, red fluorescent protein; sgRNA, single guide RNA; tf, tandem-fluorescent.
(TIF)

S3 Fig (Related to Fig 4) TMEM41B is required for autophagy. (A) Predicted topology of TMEM41B. The region of TMEM41B corresponding to pfam09335 (helices 3–5) is indicated in green. Image was generated with protter. (B) Extracts derived from wild-type HEK293T cells expressing the indicated tf construct were normalized by a BCA assay and incubated with GFP-trap beads for 1 h at 4°C. Samples were washed 5 times, eluted in 1X SDS loading buffer, and resolved by SDS-PAGE followed by IB with indicated antibodies. (C) Wild-type HEK293T cells (top) or cells expressing endogenous TMEM41B with an N-terminal GFP11 tag (bottom) were transduced with a lentivirus expressing GFP1–10 and analyzed by confocal microscopy. Shown are confocal slice micrographs of GFP fluorescence and calnexin IF, both as individual signals and merged. (D) Schematic depicting the lesions present in *TMEM41B*^*KO*^ HEK293T cells. (E) Extracts derived from wild-type and *TMEM41B*^*KO*^ HCT116 cells were resolved by SDS-PAGE followed by IB with indicated antibodies. All samples were normalized by total protein using a BCA assay prior to loading. I and II indicate the unmodified and lipidated forms of LC3. Protein levels in wild-type cells were normalized to 1. (F) Wild-type and indicated HEK293T knockout cells expressing tfSQSTM1 were analyzed by flow cytometry under basal conditions and after 18 h treatment with 100 nM BafA1 or 250 nM torin. Plots show median Red:Green ratios, inner quartiles (boxed regions), and 10th and 90th percentile (whiskers). *n* > 4,000 cells each sample. (G) Wild-type and indicated HEK293T knockout cells expressing tfLC3 were analyzed by flow cytometry under basal conditions and after 18 h treatment with 100 nM BafA1 or 250 nM torin. Plots show median Red:Green ratios, inner quartiles (boxed regions), and 10th and 90th percentile (whiskers). *n* > 1,000 cells each sample. Underlying data for all summary statistics can be found in S1 Data. BafA1, Bafilomycin A1; BCA, bicinchoninic acid; GFP, green fluorescent protein; HEK, human embryonic kidney; IB, immunoblotting; LC3, microtubule-associated protein 1 light chain 3B; SQSTM1, sequestosome 1; tf, tandem-fluorescent; TMEM41B, transmembrane protein 41B.
(TIF)

S4 Fig (Related to Fig 5) Autophagic flux is disrupted prior to phagophore maturation in the absence of *TMEM41B*. (A) Wild-type and indicated HEK293T knockout cells cell were treated with 250 nM torin for 3 h or left untreated. The corresponding cell extracts were resolved by SDS-PAGE and analyzed IB with antibodies against indicated proteins or specific phosphorylation sites. All samples were normalized by total protein using a BCA assay prior to loading, and even loading was verified by monitoring tubulin levels. I and II indicate the unmodified and lipidated forms of LC3. (B) Schematic of the protease protection assay for detecting closed autophagosomes. Lipidated LC3 (LC3-II) is indicated on the autophagosomal membrane. Dashed lines indicate proteolyzed LC3. (C) Wild-type and indicated HEK293T knockouts were treated for 18 h with BafA1 prior to gentile, mechanical lysis. The corresponding cell extracts were treated as indicated prior to being resolved by SDS-PAGE and analyzed by IB with indicated antibodies. I and II indicate unmodified and lipidated forms of LC3. (C) Quantitation of protease-protection data from experiments in B. Bar graphs show the mean ± SD of each sample from ≥4 independent experiments; *p*-values were determined using a student *t* test. **p < 0.01. Underlying data for all summary statistics can be found in S1 Data. BCA, bicinchoninic acid; HEK, human embryonic kidney; IB, immunoblotting; LC3, microtubule-associated protein 1 light chain 3B; TMEM41B, transmembrane protein 41B.
(TIF)

S5 Fig (Related to Fig 4) TMEM41B deletion arrests autophagy on-pathway prior to phagophore maturation. (A) Representative confocal micrographs (as maximum intensity projections) of wild-type and *TMEM41B*^*KO*^ HEK293T cells. Selected regions (white box) of micrographs are shown as insets of single and merged channels from IF against indicated proteins. LC3, magenta; SQSTM1, green; merged, white; Hoechst, blue. Scale bars: large panels, 5 μm; small panels, 1 μm. (B) Plots showing means of LC3^+^/SQSTM1^+^ punctae in wild-type and *TMEM41B*^*KO*^ HEK293T cells imaged in part A with inner quartiles (boxed regions), 1.5 interquartile ranges (whiskers), and outliers (dots) indicated. Sample size (*n*) for each sample is indicated. (C) Extracts derived from wild-type and indicated HEK293T single knockout and DKO (*TMEM41B*^*KO*^/*RB1CC1*^*KO*^) cells were resolved by SDS-PAGE and analyzed by IB for indicated proteins. I and II indicate unmodified and lipidated forms of LC3. Also shown are PCR results from a T7 endonuclease assay used to confirm TMEM41B gene knockouts. (D) Wild-type and indicated HEK293T knockout cells expressing GFP-STX17TM were treated with 250 nM torin for 1 h or left untreated (mock) prior to confocal microscopy. Shown are representative confocal micrographs (as maximum intensity projections). Selected regions (white boxes) of micrographs are shown as insets of single and merged channels from intrinsic GFP fluorescence or IF against indicated proteins. LC3, magenta; STX17, green; merged, white; Hoechst, blue. Scale bars: large panels, 5 μm; small panels, 1 μm. (E) Plots showing means of indicated punctae in wild-type and HEK293T knockout cells imaged in part A with inner quartiles (boxed regions), 1.5 interquartile ranges (whiskers), and outliers (dots) indicated. Sample size (*n*) for each sample is indicated. Underlying data for all summary statistics can be found in S1 Data. DKO, double knockout; GFP, green fluorescent protein; HEK, human embryonic kidney; IB, immunoblotting; IF, immunofluorescence; LC3, microtubule-associated protein 1 light chain 3B; STX17, syntaxin 17; TMEM41B, transmembrane protein 41B.
(TIF)

S6 Fig (Related to Fig 7) TEM analysis of TMEM41B^KO^ cells identifies unspecified (approximately 140 nm) vesicle accumulation and immature autophagosomal membrane intermediates. (A) Analysis of HEK293T *TMEM41B*^*KO*^ cells by TEM revealed the accumulation of approximately 140-nm, single-membrane vesicle clusters of unspecified origin. White arrowheads demarcate representative vesicle clusters. White boxes indicate region of amplification for next image in the image series. Image series demarcated by green arrows. Scale bars are indicated. (B) Alternative views of the reconstructed structure shown in Fig 7B. The spatial relationship of each image (relative to Fig 7B) is shown by the inset X, Y, Z coordinate (C) Alternative representative 3D model of a GFP+ membrane structure from HEK293T *TMEM41B*^*KO*^ cells expressing tfLC3. White arrowheads demarcate a representative structure of interest. White box indicates area of amplification for next image of the image series. Image progression demarcated by green arrows. Blue, Hoechst 33342. GFP, green fluorescent protein; HEK, human embryonic kidney; LC3, microtubule-associated protein 1 light chain 3B; TEM, transmission electron microscopy; TMEM41B, transmembrane protein 41B.
(TIF)

S7 Fig (Related to Fig 8) TMEM41B is a homolog of VMP1. (A) Alignment of Pfam09335-containing regions from Pfam09335-containing proteins in human (Hs), *Saccharomyces cerevisiae* (Sc), and *Escherichia coli* (Ec). Predicted transmembrane segments in TMEM41B are indicated as black bars. (B) Indicated tf-protein expression constructs were integrated at the AAVS1 locus in wild-type and indicated HEK293T knockout cells. Protein levels were normalized by BCA prior to resolution by SDS-PAGE and IB with indicated antibodies. Protein levels were quantified using ImageQuant. AAVS1, adeno-associated virus integration site 1; BCA, bicinchoninic acid; HEK, human embryonic kidney; IB, immunoblotting; tf, tandem-fluorescent; TMEM41B, transmembrane protein 41B; VMP1, vacuole membrane protein 1.
(TIF)

S1 Data Underlying values for all reported summary statistics. Raw data from all reported summary statistics.
(XLSX)

S2 Data Read counts from all screens. Normalized read counts from each sequencing experiment. See “read_me” tab for sample identity.
(XLSX)

S3 Data Beta scores from all screens. Individual beta scores from each experiment are listed by gene. Beta values from all replicates were averaged and are shown. Averaged values are color coded from red (suppressor) to white (neutral) to blue (enhancer). To view the underlying beta values for each replicate of a given reporter; expand the desired column by clicking the “+” at the top the column.
(XLSX)

S1 Table sgRNA oligos. List of 20-nucleotide oligos (plus overhangs) used to clone individual sgRNAs. oligo, oligonucleotide; sgRNA, single guide RNA.
(XLSX)

S2 Table Other oligos. List of oligos used in T7 endonuclease assays to confirm TMEM41B deletion and oligos used for endogenous labeling of TMEM41B with the 11th beta strand of GFP. GFP, green fluorescent protein; oligo, oligonucleotide; TMEM41B, transmembrane protein 41B.
(XLSX)

S3 Table List of primers for cloning tfReporters. List of primers used to clone LC3B and receptors into the KpnI site of tfEmpty to generate each tfReporter. LC3, microtubule-associated protein 1 light chain 3B; tf, tandem-fluorescent.
(XLSX)

S4 Table Illumina primers for Brunello library. List of primers used for Illumina sequencing of the Brunello library. Staggered oligos were pooled prior to PCR amplification. In contrast, a unique barcode primer was used for each sample. Green, Illumina P5 or P7 sequence; blue, sequencing primer annealing region; underline, stagger; red, region complementary to vector backbone (for PCR); purple, unique barcode (6-mer). oligo, oligonucleotide.
(XLSX)

## Abbreviations

AAVS1: adeno-associated virus integration site 1
ALS2: amyotrophic lateral sclerosis 2
ATG: autophagy-related; BafA1, Bafilomycin A1
BLOC-1: biogenesis of lysosome-related organelles complex 1
BORC: BLOC-one-related complex
BSD: blasticidin resistance gene
CHS: cholesteryl hemisuccinate
CLEM: correlative light and electron microscopy
COPI: coat protein I
CORVET: class C core vacuole/endosome tethering
CRISPR/Cas9: clustered regularly interspaced palindromic repeats/CRISPR-associated protein 9
DDM: n-dodecyl-β-D-maltoside
ER: endoplasmic reticulum
ESCRT: endosomal sorting complexes required for transport
FACS: fluorescence activated cell sorting
gDNA: genomic DNA
GFP: green fluorescent protein
HEK: human embryonic kidney
HOPS: homotypic fusion and vacuole protein sorting
IB: immunoblotting; IF, immunofluorescence
LC3: microtubule-associated protein 1 light chain 3B
MAGeCK: model-based analysis of genome-wide CRISPR-Cas9 knockout
NBR1: neighbor of BRCA1 gene 1
NDP52: nuclear dot protein 52
NGS: normal goat serum; oligo, oligonucleotide
p-S318: phospho-serine-318
PI3K: phosphoinositide-3 kinase
PI3P: phosphatidylinositol-3 phosphate
RB1CC1: RB1 inducible Coiled-Coil 1
RFP: red fluorescent protein
sgRNA: single guide RNA
SQSTM1: sequestosome 1
STR: Short tandem repeat
STX17: syntaxin 17
SVM: Support Vector Machine
TAX1BP1: tax1 binding protein 1
TEM: transmission electron microscopy
tf: tandem-fluorescent
TMEM41B: transmembrane protein 41B
TORC1: mammalian target of rapamycin complex 1
ULK1: Unc-51 like autophagy activating kinase 1
UROD: uroporphyrinogen decarboxylase
VMP1: vacuole membrane protein 1
VPS37A: vacuolar protein sorting 37 homolog A
WIPI2: WD repeat domain, phosphoinositide interacting 2
