# Supplementary figures and images for: CRISPR screening using an expanded toolkit of autophagy reporters identifies TMEM41B as a novel autophagy factor
Source: PLoS Biol. 2019 Apr 1;17(4):e2007044. doi: 10.1371/journal.pbio.2007044 (PMC6459555; doi:10.1371/journal.pbio.2007044)

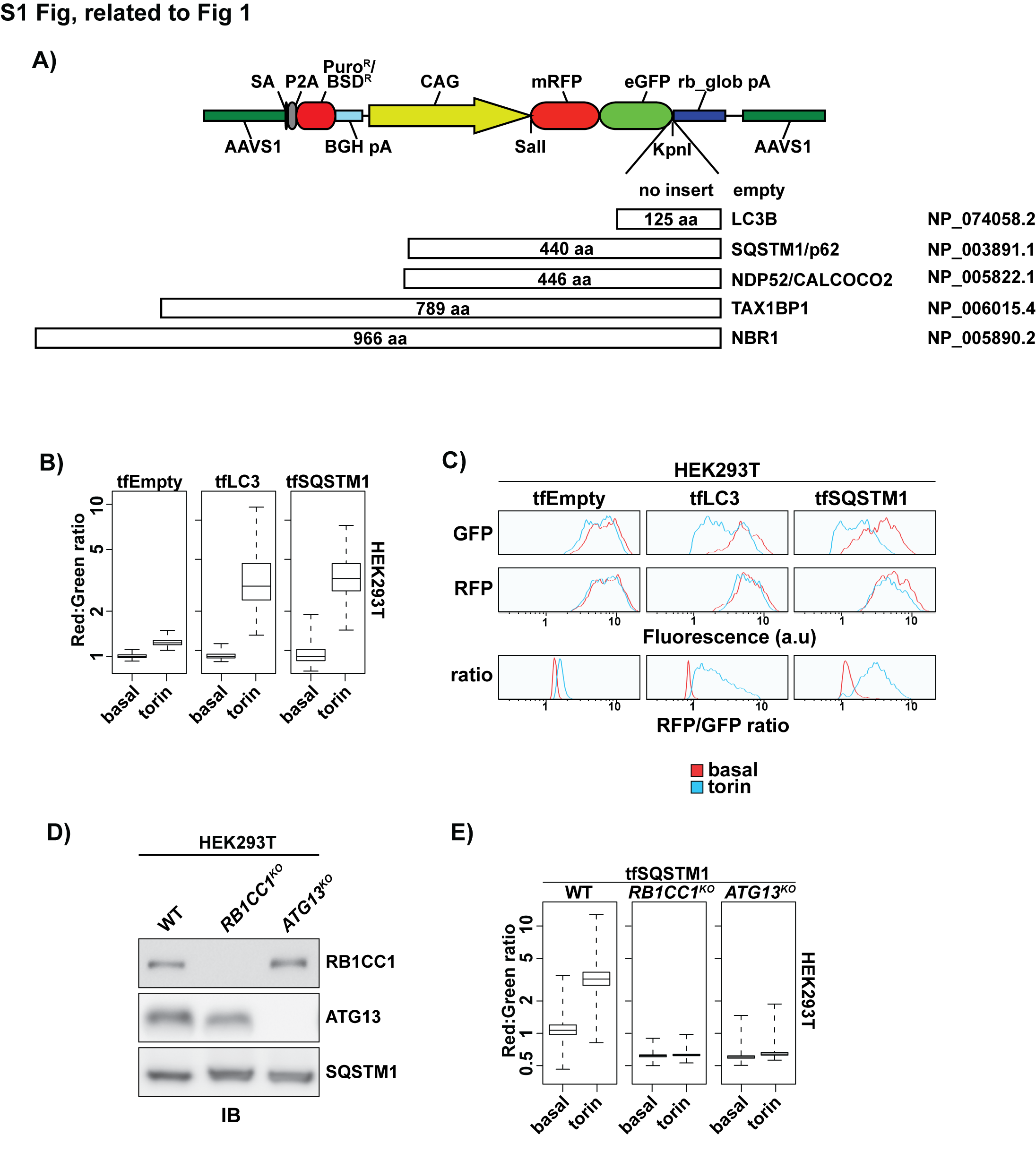

Supplement: S1 Fig — (A) Diagram of gene cassette used for expressing all tf proteins in this study. Salient cassette features are color coded. Shown below are ATG factors that were expressed as N-terminal tf fusions with their length, common names, and RefSeq accession number listed left to right. All inserts were positioned at the KpnI restriction site. (B) HEK293T cells expressing indicated tf expression cassettes from the AAVS1 locus were analyzed by flow cytometry under basal conditions and after 18 h treatment with 250 nM torin. Plots show median Red:Green ratios, inner quartiles (boxed regions), and 10th and 90th percentile (whiskers). All samples are normalized to basal Red:Green ratio. (C) HEK293T cells expressing the indicated tf proteins from the AAVS locus were grown under basal conditions or treated with torin. Shown are flow cytometry traces of GFP and RFP fluorescence (arbitrary units), both as individual signals and as a ratio (Red:Green). (D) Extracts derived from cells with indicated genotypes were normalized by total protein levels using a BCA assay and resolved by SDS-PAGE followed by IB with indicated antibodies. (E) Wild-type and indicated HEK293T knockout cells expressing tfSQSTM1 from the AAVS1 locus were treated and analyzed as in part B. Underlying data for all summary statistics can be found in S1 Data. AAVS1, AAVS homology arms; ATG, autophagy-related; BGH pA, bovine growth hormone polyadenylation signal; CAG, CAG promoter sequence; GFP, green fluorescent protein; IB, immunoblotting; P2A, self-cleaving peptide; PuroR/BSDR, puromycin or blasticidin resistance cassette; RFP, red fluorescent protein; SA, splice acceptor; tf, tandem-fluorescent. (TIF) [file pbio.2007044.s001.tif]

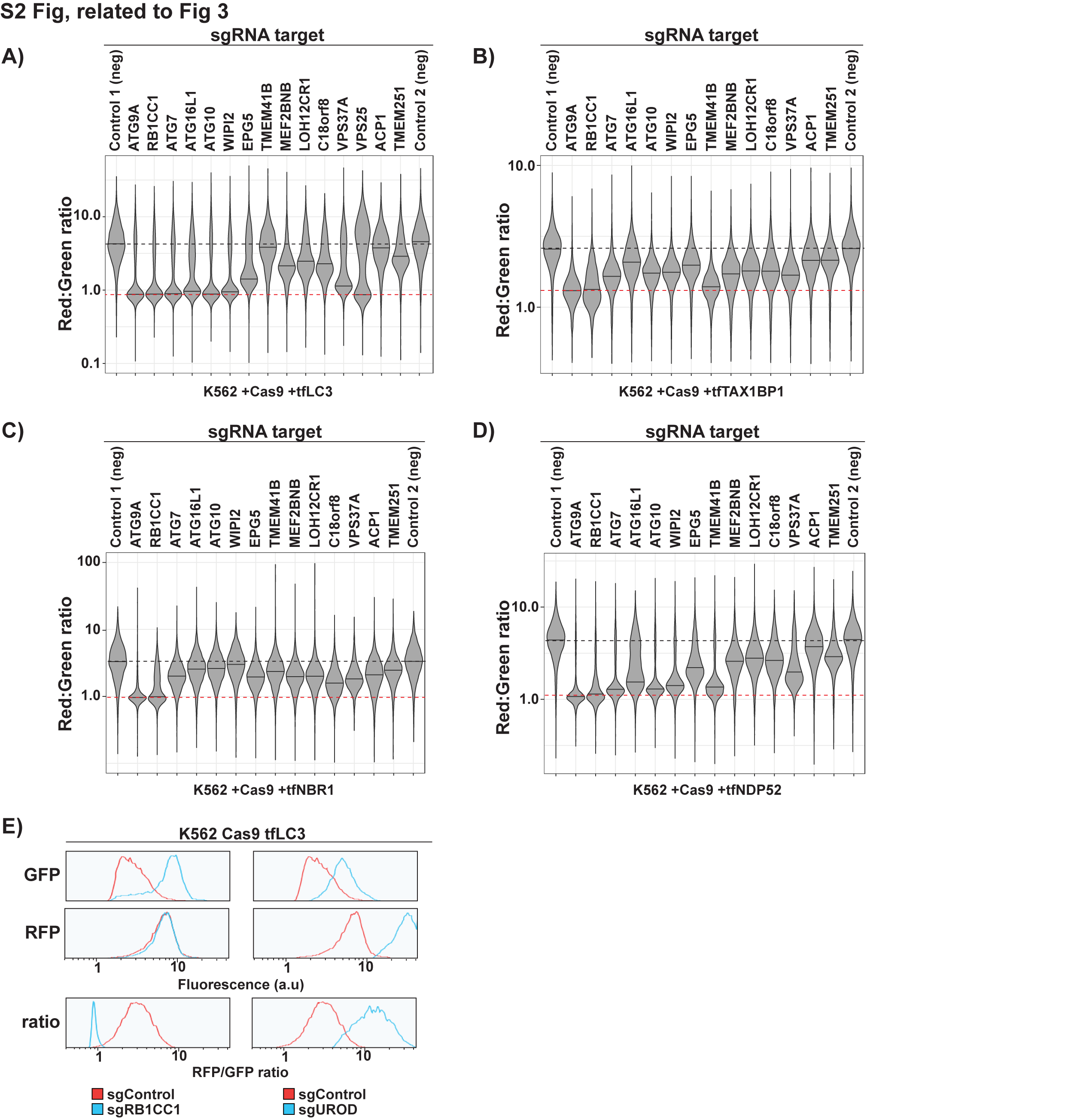

Supplement: S2 Fig — (A–D) K562 cells co-expressing Cas9 and indicated tfReporters were transduced with individual sgRNAs against the shown genes or with nontargeting sgRNA controls. Cells were treated and analyzed as in Fig 3A. These data are represented as part of the heat map in Fig 3B. n > 5,000 cells each. (E) K562 cells co-expressing Cas9 and tfLC3 were transduced with sgRNAs against the indicated genes or with a negative sgRNA control. Shown are flow cytometry traces of GFP and RFP fluorescence (in arbitrary units), both as individual signals and as a ratio (Red:Green). Cells were treated and analyzed as in panel A. Underlying data for all summary statistics can be found in S1 Data. Cas9, CRISPR-associated protein 9; GFP, green fluorescent protein; RFP, red fluorescent protein; sgRNA, single guide RNA; tf, tandem-fluorescent. (TIF) [file pbio.2007044.s002.tif]

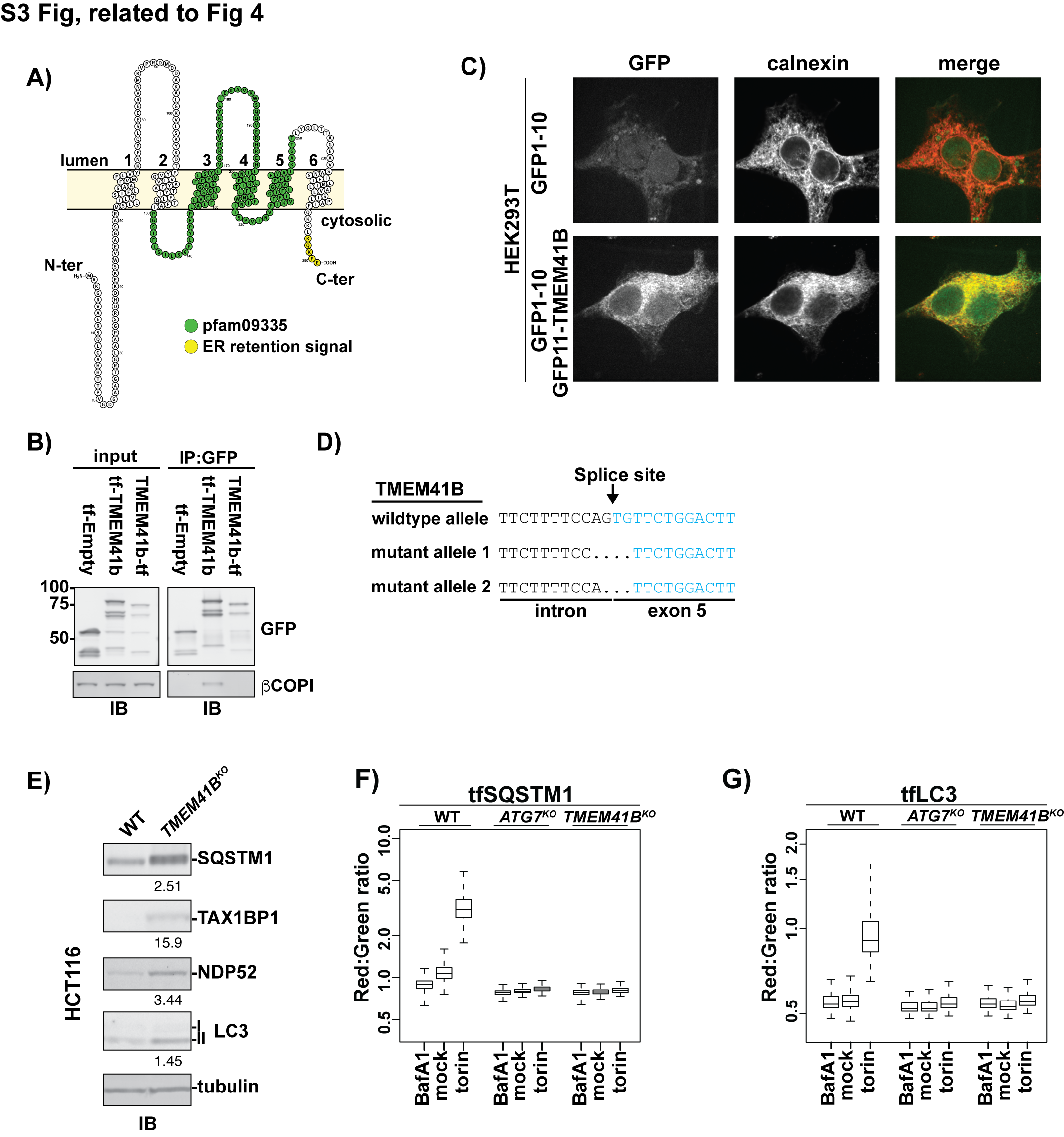

Supplement: S3 Fig — (A) Predicted topology of TMEM41B. The region of TMEM41B corresponding to pfam09335 (helices 3–5) is indicated in green. Image was generated with protter. (B) Extracts derived from wild-type HEK293T cells expressing the indicated tf construct were normalized by a BCA assay and incubated with GFP-trap beads for 1 h at 4°C. Samples were washed 5 times, eluted in 1X SDS loading buffer, and resolved by SDS-PAGE followed by IB with indicated antibodies. (C) Wild-type HEK293T cells (top) or cells expressing endogenous TMEM41B with an N-terminal GFP11 tag (bottom) were transduced with a lentivirus expressing GFP1–10 and analyzed by confocal microscopy. Shown are confocal slice micrographs of GFP fluorescence and calnexin IF, both as individual signals and merged. (D) Schematic depicting the lesions present in TMEM41BKO HEK293T cells. (E) Extracts derived from wild-type and TMEM41BKO HCT116 cells were resolved by SDS-PAGE followed by IB with indicated antibodies. All samples were normalized by total protein using a BCA assay prior to loading. I and II indicate the unmodified and lipidated forms of LC3. Protein levels in wild-type cells were normalized to 1. (F) Wild-type and indicated HEK293T knockout cells expressing tfSQSTM1 were analyzed by flow cytometry under basal conditions and after 18 h treatment with 100 nM BafA1 or 250 nM torin. Plots show median Red:Green ratios, inner quartiles (boxed regions), and 10th and 90th percentile (whiskers). n > 4,000 cells each sample. (G) Wild-type and indicated HEK293T knockout cells expressing tfLC3 were analyzed by flow cytometry under basal conditions and after 18 h treatment with 100 nM BafA1 or 250 nM torin. Plots show median Red:Green ratios, inner quartiles (boxed regions), and 10th and 90th percentile (whiskers). n > 1,000 cells each sample. Underlying data for all summary statistics can be found in S1 Data. BafA1, Bafilomycin A1; BCA, bicinchoninic acid; GFP, green fluorescent protein; HEK, human embryonic kidney; IB, immu [file pbio.2007044.s003.tif]

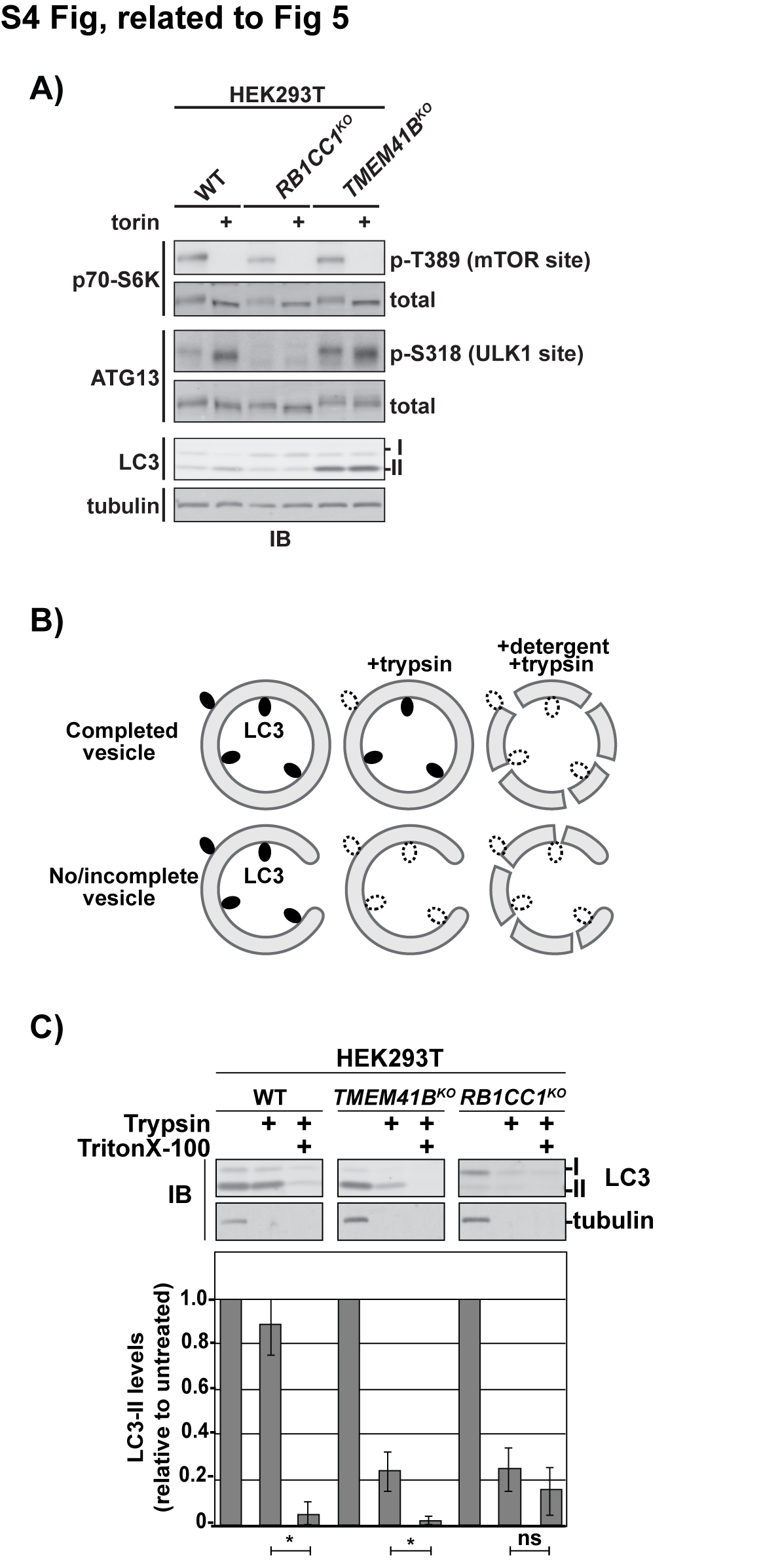

Supplement: S4 Fig — (A) Wild-type and indicated HEK293T knockout cells cell were treated with 250 nM torin for 3 h or left untreated. The corresponding cell extracts were resolved by SDS-PAGE and analyzed IB with antibodies against indicated proteins or specific phosphorylation sites. All samples were normalized by total protein using a BCA assay prior to loading, and even loading was verified by monitoring tubulin levels. I and II indicate the unmodified and lipidated forms of LC3. (B) Schematic of the protease protection assay for detecting closed autophagosomes. Lipidated LC3 (LC3-II) is indicated on the autophagosomal membrane. Dashed lines indicate proteolyzed LC3. (C) Wild-type and indicated HEK293T knockouts were treated for 18 h with BafA1 prior to gentile, mechanical lysis. The corresponding cell extracts were treated as indicated prior to being resolved by SDS-PAGE and analyzed by IB with indicated antibodies. I and II indicate unmodified and lipidated forms of LC3. (C) Quantitation of protease-protection data from experiments in B. Bar graphs show the mean ± SD of each sample from ≥4 independent experiments; p-values were determined using a student t test. **p < 0.01. Underlying data for all summary statistics can be found in S1 Data. BCA, bicinchoninic acid; HEK, human embryonic kidney; IB, immunoblotting; LC3, microtubule-associated protein 1 light chain 3B; TMEM41B, transmembrane protein 41B. (TIF) [file pbio.2007044.s004.tif]

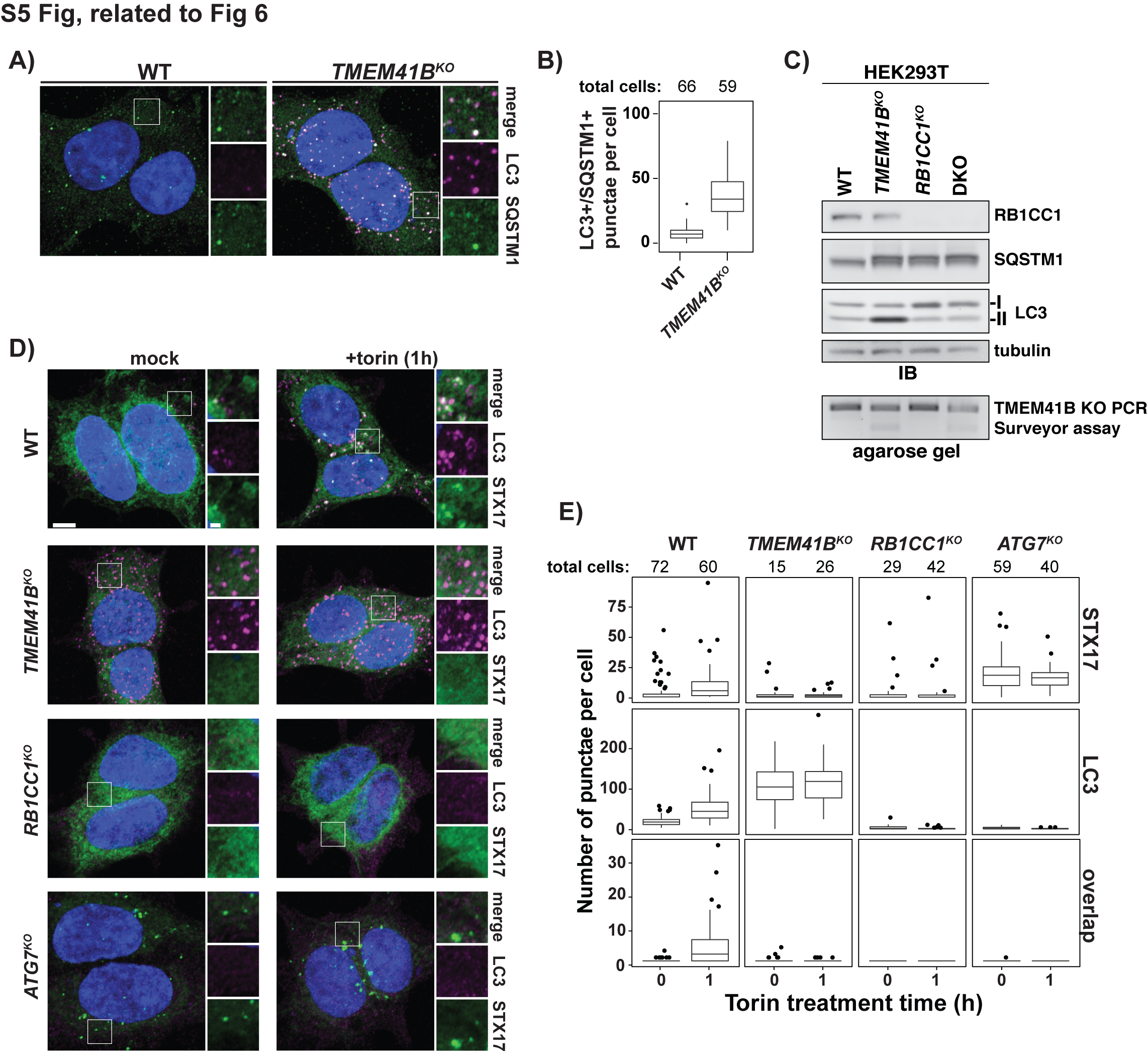

Supplement: S5 Fig — (A) Representative confocal micrographs (as maximum intensity projections) of wild-type and TMEM41BKO HEK293T cells. Selected regions (white box) of micrographs are shown as insets of single and merged channels from IF against indicated proteins. LC3, magenta; SQSTM1, green; merged, white; Hoechst, blue. Scale bars: large panels, 5 μm; small panels, 1 μm. (B) Plots showing means of LC3+/SQSTM1+ punctae in wild-type and TMEM41BKO HEK293T cells imaged in part A with inner quartiles (boxed regions), 1.5 interquartile ranges (whiskers), and outliers (dots) indicated. Sample size (n) for each sample is indicated. (C) Extracts derived from wild-type and indicated HEK293T single knockout and DKO (TMEM41BKO/RB1CC1KO) cells were resolved by SDS-PAGE and analyzed by IB for indicated proteins. I and II indicate unmodified and lipidated forms of LC3. Also shown are PCR results from a T7 endonuclease assay used to confirm TMEM41B gene knockouts. (D) Wild-type and indicated HEK293T knockout cells expressing GFP-STX17TM were treated with 250 nM torin for 1 h or left untreated (mock) prior to confocal microscopy. Shown are representative confocal micrographs (as maximum intensity projections). Selected regions (white boxes) of micrographs are shown as insets of single and merged channels from intrinsic GFP fluorescence or IF against indicated proteins. LC3, magenta; STX17, green; merged, white; Hoechst, blue. Scale bars: large panels, 5 μm; small panels, 1 μm. (E) Plots showing means of indicated punctae in wild-type and HEK293T knockout cells imaged in part A with inner quartiles (boxed regions), 1.5 interquartile ranges (whiskers), and outliers (dots) indicated. Sample size (n) for each sample is indicated. Underlying data for all summary statistics can be found in S1 Data. DKO, double knockout; GFP, green fluorescent protein; HEK, human embryonic kidney; IB, immunoblotting; IF, immunofluorescence; LC3, microtubule-associated protein 1 light chain 3B; STX17, syntaxin 17; TMEM41B, [file pbio.2007044.s005.tif]

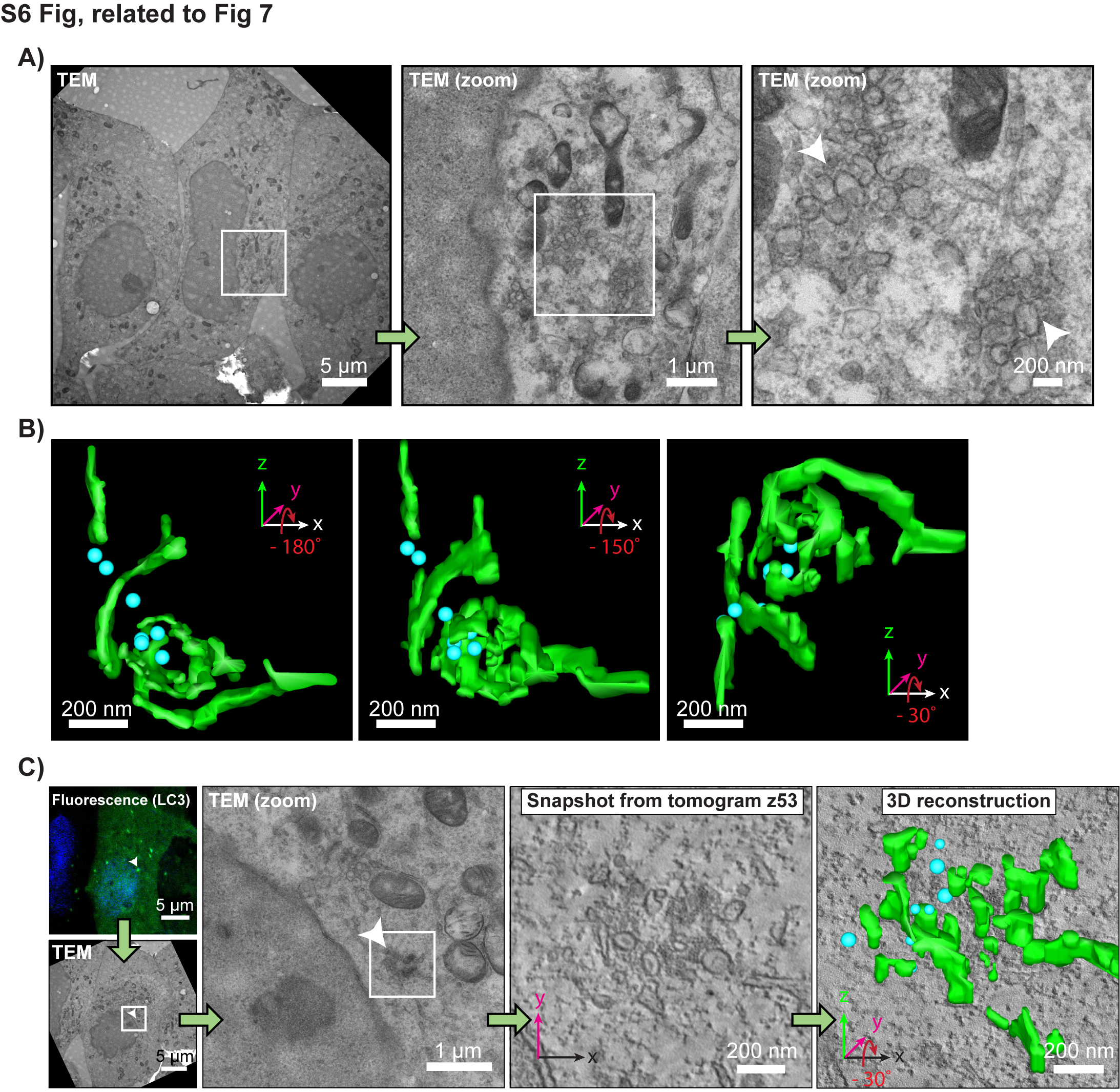

Supplement: S6 Fig — (A) Analysis of HEK293T TMEM41BKO cells by TEM revealed the accumulation of approximately 140-nm, single-membrane vesicle clusters of unspecified origin. White arrowheads demarcate representative vesicle clusters. White boxes indicate region of amplification for next image in the image series. Image series demarcated by green arrows. Scale bars are indicated. (B) Alternative views of the reconstructed structure shown in Fig 7B. The spatial relationship of each image (relative to Fig 7B) is shown by the inset X, Y, Z coordinate (C) Alternative representative 3D model of a GFP+ membrane structure from HEK293T TMEM41BKO cells expressing tfLC3. White arrowheads demarcate a representative structure of interest. White box indicates area of amplification for next image of the image series. Image progression demarcated by green arrows. Blue, Hoechst 33342. GFP, green fluorescent protein; HEK, human embryonic kidney; LC3, microtubule-associated protein 1 light chain 3B; TEM, transmission electron microscopy; TMEM41B, transmembrane protein 41B. (TIF) [file pbio.2007044.s006.tif]

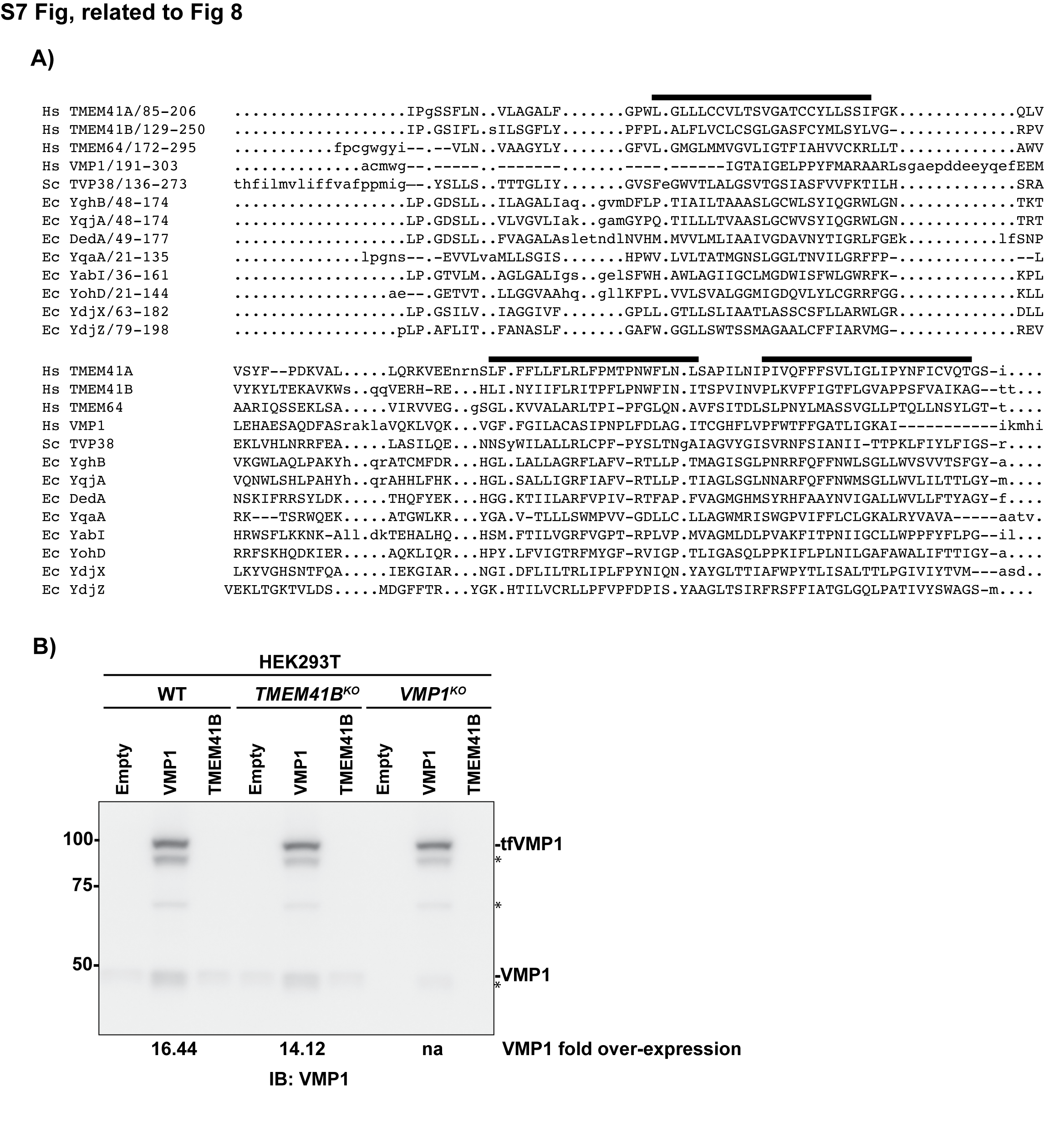

Supplement: S7 Fig — (A) Alignment of Pfam09335-containing regions from Pfam09335-containing proteins in human (Hs), Saccharomyces cerevisiae (Sc), and Escherichia coli (Ec). Predicted transmembrane segments in TMEM41B are indicated as black bars. (B) Indicated tf-protein expression constructs were integrated at the AAVS1 locus in wild-type and indicated HEK293T knockout cells. Protein levels were normalized by BCA prior to resolution by SDS-PAGE and IB with indicated antibodies. Protein levels were quantified using ImageQuant. AAVS1, adeno-associated virus integration site 1; BCA, bicinchoninic acid; HEK, human embryonic kidney; IB, immunoblotting; tf, tandem-fluorescent; TMEM41B, transmembrane protein 41B; VMP1, vacuole membrane protein 1. (TIF) [file pbio.2007044.s007.tif]
